# Joint Effect of *MCP-1* Genotype *GG* and *MMP-1* Genotype *2G/2G* Increases the Likelihood of Developing Pulmonary Tuberculosis in BCG-Vaccinated Individuals

**DOI:** 10.1371/journal.pone.0008881

**Published:** 2010-01-25

**Authors:** Malathesha Ganachari, Jorge A. Ruiz-Morales, Juan C. Gomez de la Torre Pretell, Jeffrey Dinh, Julio Granados, Pedro O. Flores-Villanueva

**Affiliations:** 1 The Methodist Hospital Research Institute, The Methodist Hospital, Houston, Texas, United States of America; 2 Non-Governmental Organization VIMASI, San Borja, Lima, Perú; 3 Division of Immunogenetics, Department of Transplantation Immunology, Mexican National Institute of Medicine and Nutrition “Salvador Zubiran”, Mexico D.F., Mexico; Instituto Gulbenkian de Ciência, Portugal

## Abstract

We previously reported that the – 2518 *MCP*-1 genotype *GG* increases the likelihood of developing tuberculosis (TB) in non-BCG-vaccinated Mexicans and Koreans. Here, we tested the hypothesis that this genotype, alone or together with the – 1607 *MMP-1* functional polymorphism, increases the likelihood of developing TB in BCG-vaccinated individuals. We conducted population-based case-control studies of BCG-vaccinated individuals in Mexico and Peru that included 193 TB cases and 243 healthy tuberculin-positive controls from Mexico and 701 TB cases and 796 controls from Peru. We also performed immunohistochemistry (IHC) analysis of lymph nodes from carriers of relevant two-locus genotypes and *in vitro* studies to determine how these variants may operate to increase the risk of developing active disease. We report that a joint effect between the – 2518 *MCP-1* genotype *GG* and the – 1607 *MMP-1* genotype *2G/2G* consistently increases the odds of developing TB 3.59-fold in Mexicans and 3.9-fold in Peruvians. IHC analysis of lymph nodes indicated that carriers of the two-locus genotype *MCP-1 GG MMP-1 2G/2G* express the highest levels of both MCP-1 and MMP-1. Carriers of these susceptibility genotypes might be at increased risk of developing TB because they produce high levels of MCP-1, which enhances the induction of MMP-1 production by *M. tuberculosis-*sonicate antigens to higher levels than in carriers of the other two-locus *MCP-1 MMP-1* genotypes studied. This notion was supported by *in vitro* experiments and luciferase based promoter activity assay. MMP-1 may destabilize granuloma formation and promote tissue damage and disease progression early in the infection. Our findings may foster the development of new and personalized therapeutic approaches targeting MCP-1 and/or MMP-1.

## Introduction

We previously reported an association between the -2518 functional promoter polymorphism (-*2518A>G*) of the *Monocyte Chemoattractant Protein* (*MCP*)-1 gene and susceptibility to pulmonary tuberculosis (TB) in non BCG-vaccinated Mexicans and Koreans [1]. The chromosome region 17q11.2, where the *MCP-1* gene is located, was identified as a TB susceptibility region by linkage analysis of multi-case families [2]. Fine mapping of this region using linkage disequilibrium association mapping and potentially functional SNPs revealed association of the -2518 *MCP-1* SNP the with disease [1]. Given that most of individuals living in endemic countries are vaccinated with BCG, we wondered whether the – 2518 *MCP-1* susceptibility allele *G* and *GG* genotype also increase the odds of developing disease in BCG-vaccinated individuals.

The *Matrix Metalloproteinase* (*MMP*)*-1* gene, which encodes a potent collagenase, is an excellent candidate gene for influencing the expression of pulmonary TB since inflammation-mediated tissue damage may contribute to the spread of *M. tuberculosis* infection [3], [4]. Persistent degradation of the extracellular matrix (ECM) during inflammation leads to tissue damage [3], [4]. Collagen is an essential component of lung ECM and of *M. tuberculosis*-induced granulomas [5], [6] and provides support to macrophages and T-cells, which play a role in the organization of these immune-mediated granulomas [6], [7]. In the context of granulomatous inflammation, the collagenase activity of MMP-1 is involved in inflammation-induced tissue damage in several lung diseases [8] and may contribute to the destabilization of granuloma formation and the damage of adjacent tissues in TB [9], [10]. As granulomas in TB prevent the spread of infection, MMP-1 may contribute to the dissemination of infection and to disease progression [7]. A functional polymorphism located in the promoter region of *MMP-1* enhances expression of the gene [11]. This polymorphism consists of the insertion of a guanine at position – 1607 (-*1607_1608insG*) and creates an Ets-1 transcription factor binding site [11]. Furthermore, a genome wide analysis showed that the chromosome region 11q14–11q23 may contribute to the regulation of TNF-alpha in TB, and the *MMP-1* gene is located within that region at 11q22.2 [12].

Given that both the -2518 *MCP-1* allele *G* and the -1607 *MMP-1* allele *2G* increase expression of the genes, and MCP-1 contributes to ECM remodeling through the induction of *MMP-1* expression in bronchial epithelial cells [1], [13], [14], we tested the hypothesis that these biologically functional polymorphisms, alone or in combination, might influence the expression of phenotypes prone to disease progression.

We selected Mexicans and Peruvians for this study because they have similar ethnic backgrounds but are exposed to different environmental conditions. Moreover, the TB burden is considered to be low in Mexico (0 to 24 estimated new TB cases per 100,000 population), but moderate to high in Peru (100 to 299 estimated new TB cases per 100,000 population) [15].

## Results

### Clinical Features of Cases and Controls

All study subjects were vaccinated with BCG at birth. We studied 193 and 701 sputum smear-positive cases of TB from Mexico and Peru, respectively (Table 1). None of the patients had history of any preconditions that may increase the chance of developing active TB, including preconditions affecting immune function, malnutrition and other lung diseases. Their body mass indexes (BMI) were ≥18.5 kg/m^2^ before disease onset (Table 1). In addition, all cases were new TB cases: none had a history of previous TB or had received previous treatment for TB. Thus, these TB patients were very unlikely to be cases of re-infection or recidivating TB. The patients developed evident symptoms of disease less than 1 year after exposure to a TB case. All patients had active TB disease of at least three months of evolution with chest radiographs showing alveolar infiltrates in the lungs. Hilar adenopathy was present in most Mexican (98%) and Peruvian cases (96%). None of the patients had multi-drug resistant (MDR)-TB.

**Table 1:** Demographic and clinical features of tuberculosis cases and controls A.

Parameter	Mexicans		Peruvians	
	Tuberculosis(n = 193)	Healthy Tuberculin-Positive(n = 243)	Tuberculosis(n = 701)	Healthy Tuberculin-Positive(n = 796)
Gender				
Male, n (%)	125 (65)	153 (63)	404 (57.6)	440 (55.3)
Age (years), mean ± SD				
Female	35±7	36±3	29±10	33±9
Male	36±4	37±2	30±10	34±9
BMI B (kg/m^2^), mean ± SD				
Female	26.2±5	27.3±5.6	22.8±3.2	25.2±4.5
Male	25.4±3	25.5±4.4	22.7±3.2	25.3±3.7
Smokers, n (%) C				
Female	5 (2.6)	4 (1.6)	5 (0.01)	29 (0.036)
Male	17 (8.8)	22 (9)	46 (0.07)	35 (0.044)
Alcohol use, n (%) D				
Female	0 (0)	0 (0)	0 (0)	0 (0)
Male	13 (6.7)	21 (8.6)	24 (0.034)	13 (0.016)

A The distribution of categorical variables was compared by *x^2^* or Fisher's exact tests and that of continuous variables by student t-test. There were no significant differences between the groups for any of the parameters tested.

B BMI  =  body mass index, which was based on self-report of weight, before development of disease in the case of tuberculosis patients, and height measured by a nurse.

C All smokers reported consumption of <6 cigarettes per day.

D All alcohol users reported consumption of <1 drink per day.

We also studied 243 and 796 healthy tuberculin-positive (PPD+) control individuals in Mexico and Peru, respectively (Table 1). Approximately 91% of Mexican and 98% of Peruvian controls were household contacts of a sputum smear-positive TB case, the rest were close contacts of community cases. All controls showed a PPD response >15 mm of induration and remained healthy for a period of more than 2 years after exposure, making it likely that they were latently infected individuals with capacity to control the spread of infection during that critical period [16]–[20].

All cases and controls were negative in serologic tests for HIV infection. TB cases and controls were similar in demographics, BMI (prior to development of disease in the case of TB patients), and consumption of cigarettes and alcohol, both within and between populations (Table 1). We did not have a significant proportion of individuals with these habits among our cases or controls in either of the populations studied. Cases and controls were of similar socioeconomic status within nationalities and all individuals recruited were of low socioeconomic status. We attained successful genotyping rates in our cases and controls from Mexico and Peru.

Mexican and Peruvian cases and controls were Mestizo to the third generation. Mestizos from Mexico and Peru are mainly an admixture of Spanish, Amerindians and Africans. None of our cases or controls were pure Amerindians, neither pure Caucasians nor pure non-Caucasian Africans. Thus, the presence of sub-populations in our samples of cases or controls from Peru and Mexico is unlikely. Mexican Mestizos from Mexico D.F. (the Federal District) have an admixture of Spanish (50.03±4.11%), Amerindian (49.03±3.76%), and African (0.94±1.27%) [21]. The genetic background of Peruvian Mestizo is close to that of Mexican Mestizo [22]; however, since subtle differences in the admixture of cases and controls may introduce bias we applied a methodology developed by Reich and Golstein to adjust for differences in the genetic admixture of cases and controls as explained in Materials and Methods [23]. The results of these corrections are provided in Table 2 and discussed in the next section.

**Table 2:** Univariate analysis of *MCP-1* and *MMP-1* alleles and progression to pulmonary tuberculosis A.

***Mexicans***	**TB** B(386 chromosomes)n (frequency)	**PPD+** C(486 chromosomes)n (frequency)	**TB vs PPD+**OR (95% CI) D(uncorrected statistics)	**TB vs PPD+**Corrected statistics
*- 2518 MCP-1*				
A	123 (0.32)	267 (0.55)	1.75 (1.3–2.3)	*x* ^2^/λ = 11, p = 0.0041
G	263 (0.68)	219 (0.45)	(*x* ^2^ = 15.72, p = 0.0001)	*p = 0.0082
*- 1607 MMP-1*				
1G	73 (0.19)	179 (0.27)	1.56 (1.14–2.16)	*x* ^2^/λ = 6.27, p = 0.012
2G	313 (0.81)	489 (0.73)	(*x* ^2^ = 8.36, p = 0.0038)	*p = 0.024

*Peruvians*	TB B **(1402 chromosomes)** **n (frequency)**	PPD+ C **(1592 chromosomes)** **n (frequency)**	TB vs PPD+**OR (95% CI) D** **(uncorrected statistics)**	TB vs PPD+**Corrected statistics**
*- 2518 MCP-1*				
A	421 (0.3)	567 (0.36)	1.29 (1.1–1.5)	*x* ^2^/λ = 8.54, *p* = 0.0035
G	981 (0.7)	1025 (0.64)	(*x* ^2^ = 10.52, *p* = 0.0012)	*p = 0.007
*- 1607 MMP-1*				
1G	313 (0.22)	460 (0.29)	1.4 (1.19–1.67)	*x* ^2^/λ = 13.64, *p* = 0.0002
2G	1089 (0.78)	1132 (0.71)	(*x* ^2^ = 16.8, *p* = 0.00001)	*p = 0.0004

A Groups were compared by *x^2^* analysis: *x^2^* values obtained were first corrected for population admixture dividing *x^2^* by *λ* (genomic controls coefficient λ** = **1.332 for Mexicans and  = 1.2315 for Peruvians), and the *p-*values corrected according to the number of comparisons (*p  =  Bonferroni corrected). Two comparisons were done in Mexicans and Peruvians.

B TB  =  New cases of pulmonary TB.

C PPD+  =  Healthy tuberculin reactors.

D OR  =  Odds ratio, CI  =  Confidence interval.

Based on the frequency of alleles at the *MCP-1* and *MMP-1* loci in both populations, we estimated that given the size of both samples we had 90% power to detect an odds ratio (OR) of 1.5, which was significant at an α of 0.025. Considering all the characteristics of our samples and our genotyping success rate, it is unlikely that we obtained our results because of selection bias (including poor ascertainment of the phenotypes) or information bias (including genotyping errors), or due to an unadjusted known confounder or type I and II errors.

### Univariate Analysis of the -2518 *MCP-1* and -1607 *MMP-1* Promoter Polymorphisms and Susceptibility to TB in BCG-Vaccinated Individuals

The distribution of *MCP-1* and *MMP-1* genotypes in Mexican and Peruvian controls were in Hardy-Weinberg equilibrium. The genomic control SNPs were all in Hardy-Weinberg equilibrium (Table S1) [1]. We observed a significant association of the *MCP-1* susceptibility allele *G* with disease progression in Both Mexicans (OR = 1.75; 95% CI 1.3–2.3) and Peruvians (OR = 1.29; 95% CI 1.1–1.5) (Table 2). This association remained significant after correction for genetic admixture and for the number of comparisons in both Mexicans and Peruvians (Table 2). The *MCP-1* genotype *GG* was significantly associated with TB in Mexicans (OR = 2.66, 95% CI 1.47–4.79, *p* = 0.001, Table 3) and marginally associated with disease in Peruvians (OR = 1.43, 95% CI 1.02–2.0, *p* = 0.036, Table 4).

**Table 3:** Univariate analysis of *MCP-1* and *MMP-1* genotypes and progression to pulmonary tuberculosis in Mexicans.

***MCP-1*** ** genotypes** A	**TB** B(n = 193)n (frequency)	**PPD+** C(n = 243)n (frequency)	**TB vs. PPD+**OR (95% CI) ^D^	*x* ^2^	*p value*
*AA*	23 (0.12)	46 (0.19)	1.0		
*AG*	77 (0.4)	127 (0.52)	1.21 (0.68–2.15)	0.43	0.5
*GG*	93 (0.48)	70 (0.29)	2.66 (1.47–4.79)	10.87	0.001
			Test of homogeneity (equal odds): *x* ^2^ = 17.62, *p* = 0.0001		
***MMP-1*** ** genotypes** A	(n = 193)n (frequency)	(n = 243)n (frequency)	OR (95% CI) D	*x* ^2^	*p value*
*1G/1G*	8 (0.04)	20 (0.08)	1.0		
*1G/2G*	57 (0.3)	105 (0.43)	1.36 (0.56–3.27)	0.5	0.5
*2G/2G*	128 (0.66)	118 (0.49)	2.71 (1.15–6.3)	5.51	0.02
			Test of homogeneity (equal odds): *x* ^2^ = 14.19, *p* = 0.0002		

A Groups were compared by *x^2^* Mantel-Haenszel statistics with genotypes arranged in an ordinal scale.

B TB  =  New cases of pulmonary TB.

C PPD+  =  Healthy tuberculin reactors.

D OR  =  Odds ratio, CI  =  Confidence interval.

**Table 4:** Univariate analysis of *MCP-1* and *MMP-1* genotypes and progression to pulmonary tuberculosis in Peruvians.

***MCP-1*** ** genotypes** A	**TB** B(n = 701)n (frequency)	**PPD+** C(n = 796)n (frequency)	**TB vs. PPD+**OR (95% CI) ^D^	*x* ^2^	*p value*
*AA*	74 (0.11)	98 (0.123)	1.0		
*AG*	273 (0.39)	371 (0.465)	0.97 (0.69–1.369)	0.02	0.88
*GG*	354 (0.5)	327 (0.412)	1.43 (1.02–2.0)	4.4	0.036
			Test of homogeneity (equal odds): *x* ^2^ = 13.35, *p* = 0.0013		
***MMP-1*** ** genotypes** A	(n = 701)n (frequency)	(n = 796)n (frequency)	OR (95% CI) D	*x* ^2^	*p value*
*1G/1G*	42 (0.06)	62 (0.08)	1.0		
*1G/2G*	229 (0.33)	336 (0.42)	1.0 (0.65–1.54)	0.00	0.98
*2G/2G*	430 (0.61)	398 (0.5)	1.6 (1.05–2.4)	4.92	0.027
			Test of homogeneity (equal odds): *x* ^2^ = 19.38, *p* = 0.0001		

A Groups were compared by *x^2^* Mantel-Haenszel statistics with genotypes arranged in an ordinal scale.

B TB  =  New cases of pulmonary TB.

C PPD+  =  Healthy tuberculin reactors.

D OR  =  Odds ratio, CI  =  Confidence interval.

We noticed that our current point estimates of the -2518 *MCP-1* allele *G* and genotype *GG* associations with disease were lower than those previously published for individuals non-vaccinated with the BCG [1]. Thus, we pooled the information from Mexican cases and controls recruited for the previous and present studies. We observed an almost 2-fold drop in the OR for the association of the -2518 *MCP-1* genotype *GG* with disease progression in BCG-vaccinated individuals (OR = 2.66) when compared with the total Mexican sample (OR = 4.2) or non-vaccinated individuals (OR = 5.4) (Figure S1). In addition, the dose effect of the allele *G* observed in non-vaccinated was lost in BCG-vaccinated individuals and we did not see a significant association of the *MCP-1* genotype *AG* with disease in this stratum. Given that the odds of developing active disease (reflected by the measured OR) were not homogeneous in strata formed by BCG vaccination status (Figure S1), we concluded that BCG vaccination is a modifier of the effect of the *MCP-1* susceptibility allele *G* and genotype *GG* on progression to disease [24].

Our results from analysis of the -1607 *MMP-1* polymorphism revealed that this polymorphism influences the expression of active disease in both, Mexican and Peruvian populations. Although the point estimates of main effects (ORs) were limited in strength, we observed an association of the -1607 *MMP-1* allele *2G* with susceptibility to developing TB in both Mexicans and Peruvians (Table 2). The proportion of carriers of the *MMP-1* allele *2G* was increased among Mexican (OR = 1.56. 95% CI 1.14–2.16, Table 2) and Peruvian (OR = 1.4, 95% CI 1.19–1.67, Table 2) TB cases. This association remained significant after corrections for population stratification and number of comparisons. Carriers of the *MMP-1* genotype *2G/2G* were significantly over-represented in Mexican TB cases (OR = 2.71, 95% CI 1.15–6.3, *p* = 0.02, Table 3) and in Peruvian TB cases (OR = 1.6, 95% CI 1.05–2.4, *p* = 0.027, Table 4).

The population admixture coefficients (λ) calculated for Mexicans (1.332) and Peruvians (1.2315) were low and did not significantly modify our estimates of associations of alleles with disease (Table S1 and Table 2) [1]. We did not see major differences in the admixture background of our cases and controls, or these differences were not significant enough to bias our analysis of associations [23].

### Multivariate Analysis of Main and Joint Effects of the *-2518 MCP-1* and -1607 *MMP-1* Genotypes in Susceptibility to Developing Active Pulmonary TB

Based on the results obtained in the univariate analysis of associations we collapsed *MCP-1* and *MMP-1* genotypes that were not found to be associated with TB in both populations. Thus, the *MCP-1* genotypes *AA* and *AG* and *MMP-1* genotypes *1G/1G* and *1G/2G* were collapsed into *MCP-1* genotype *A/-* and *MMP-1* genotype *1G/-,* respectively. Collapsing non-significant genotypes at each locus allowed us to test the hypothesis that the joint effects of susceptibility genotypes -2518 *MCP-1 GG* and -1607 *MMP-1 2G/2G* are greater than that of the independent (main) effects of these two genotypes. We obtained a significant and consistent joint effect of the two-locus genotype *MCP-1 GG* and *MMP-1 2G/2G* in both Mexicans and Peruvians. The ORs observed in carriers of the two-locus genotype *MCP-1 GG* and *MMP-1 2G/2G* were 3.59 (95% CI 1.54–8.33, *p* = 0.003, Table 5) in Mexicans and 3.9 (95% CI 2.56–5.95, *p* = 0.0001, Table 6) in Peruvians. In Peruvians, the logistic regression adjusted OR estimated for the carriers of the *MCP-1* genotype *GG* that did not carry the *MMP-1* genotype *2G/2G* was 0.69 (*p* = *0.02*). This was because most of the Peruvian cases carrying the *MCP-1* genotype *GG* also carried the *MMP-1* genotype *2G/2G* (Table 6).

**Table 5:** Multivariate analysis of main and joint effects of *MCP-1* and *MMP-1* tuberculosis susceptibility genotypes in Mexicans.

**Mexicans** A	**TB** B(n = 193)n (frequency)	**PPD+** C(n = 243)n (frequency)			
*A/- 1G/-*	45 (0.23)	86 (0.35)			
*A/- 2G/2G*	55 (0.28)	87 (0.36)			
*GG 1G/-*	20 (0.10)	38 (0.16)			
*GG 2G/2G*	73 (0.38)	32 (0.13)			
		**TB versus PPD+**			
		OR (95% CI) D	Standard error	z	*p value*
Main effect of *MMP-1* genotype *2G/2G*		1.21 (0.73–1.98)	0.75	0.75	0.45
Main effect of *MCP-1* genotype *GG*		1.00 (0.52–1.93)	0.33	0.02	0.98
Joint effects of *MMP-1 2G/2G* and *MCP-1 GG*		3.59 (1.54–8.33)	1.54	2.97	0.003

A Groups were compared using multiple logistic regression analysis. In the annotations for combinations of genotype at the two loci under study, the *MCP-1* genotype is listed first followed by the *MMP-1* genotype. Individuals carrying *MCP-1* genotypes *AA* or *AG* were grouped under the *A/-* denomination. Individuals carrying *MMP-1* genotypes *1G/1G* or *1G/2G* were grouped under *1G/-* denomination. The following is an example of annotations of combination genotypes at the two loci studied: *A/- 1G/-* refers to individual carriers of a genotype *AA* or *AG* at the *MCP-1* locus *and* a genotype *1G/1G* or *1G/2G* at *MMP-1* locus.

B TB  =  New cases of pulmonary TB.

C PPD+  =  Healthy tuberculin reactors.

D OR  =  Odds ratio, CI  =  Confidence interval.

**Table 6:** Multivariate analysis of main and joint effects of *MCP-1* and *MMP-1* tuberculosis susceptibility genotypes in Peruvians.

**Peruvians** A	**TB** B(n = 701)n (frequency)	**PPD+** C(n = 796)n (frequency)			
*A/- 1G/-*	163 (0.23)	203 (0.26)			
*A/- 2G/2G*	184 (0.26)	266 (0.33)			
*GG 1G/-*	108 (0.15)	195 (0.24)			
*GG 2G/2G*	246 (0.35)	132 (0.17)			
		**TB versus PPD+**			
		OR (95% CI) D	Standard error	z	p value
Main effect of *MMP-1* genotype *2G/2G*		0.86 (0.65–1.14)	0.12	−1.05	0.295
Main effect of *MCP-1* genotype *GG*		0.69 (0.5–0.94)	0.11	−2.33	0.02
Joint effects of *MMP-1* 2G/2G and *MCP-1 GG*		3.9 (2.56–5.95)	0.84	6.33	0.0001

A Groups were compared using multiple logistic regression analysis. In the annotations for combinations of genotype at the two loci under study, the *MCP-1* genotype is listed first followed by the *MMP-1* genotype. Individuals carrying *MCP-1* genotypes *AA* or *AG* were grouped under the *A/-* denomination. Individuals carrying *MMP-1* genotypes *1G/1G* or *1G/2G* were grouped under the *1G/-* denomination. The following is an example of annotations of combination genotypes at the two loci studied: *A/- 1G/-* refers to individual carriers of a genotype *AA* or *AG* at the *MCP-1* locus *and* a genotype *1G/1G* or *1G/2G* at *MMP-1* locus.

B TB  =  New cases of pulmonary TB.

C PPD+  =  Healthy tuberculin reactors.

D OR  =  Odds ratio, CI  =  Confidence interval.

We used the Pearson goodness-of-fit *x* ^2^ and the Hosmer-Lemeshow (HL) *x* ^2^ tests to estimate the magnitude of the differences between the event rate predicted by the logistic models and that observed in the cases and controls to which the models were applied [24], [25]. The null hypothesis for these tests states that the expected and observed event rates predicted by the model are not significantly different. A *p*-value >0.1 indicates that the model provides a good fit for the data since at this level of significance the null hypothesis cannot be rejected [24], [25]. The full model (main and interaction terms) and the interaction model alone fitted the observed data. Indeed, the statistic estimates (*x* ^2^ and *p* values) were the same for the full and the interaction models in Mexicans and Peruvians (full model: Pearson *x* ^2^ = 0, *p*>0.1 and HL *x* ^2^ = 0, *p*>0.1*;* interaction model: Pearson *x* ^2^ = 0, *p*>0.1 and HL *x* ^2^ = 0, *p*>0.1). These results clearly indicate that the interaction model alone was sufficient and provides a better fit for the observed data. Consistent with this observations, a model with the main effects alone was not sufficient to explain the data observed and the null hypothesis was rejected in Mexicans (Pearson *x* ^2^ = 8.95, *p* = 0.0028; HL *x* ^2^ = 8.95, *p* = 0.0114) or Peruvians (Pearson *x* ^2^ = 40.56, *p*<0.00001; HL *x* ^2^ = 17.83, *p*<0.00001). In addition, the ORs obtained for the joint effects were greater than the expected assuming additivity in both populations. Indeed, the ORs calculated for the additive model were 1.21 and 0.55 in Mexicans and Peruvians, respectively (Tables 5 and 6, Materials and Methods section) [24]. Thus, we concluded that the *MCP-1* and *MMP-1* genotypes *GG* and *2G/2G* jointly increase the odds of developing TB in Mexicans and Peruvians, and that these joint effects are multiplicative [24].

### Immunohistochemistry (IHC) Analysis of Lymph Nodes from Tuberculosis Cases Carriers of Relevant *MCP-1* and *MMP-1* Genotypes

Early events are relevant for progression from infection to disease [1], [26]. Consequently, we analyzed lymph nodes from untreated Peruvian TB cases that were experiencing a local (node) inflammatory process with less than 15 days of evolution. We assessed whether carriers of the *MCP-1* genotype *GG* and *MMP-1* genotype *2G/2G* produce increased levels of MCP-1 and MMP-1 at an early stage of the inflammatory process [1], [26]. We used immunohistochemistry (IHC) since the available specimens were archived samples fixed in paraformaldehyde and embedded in paraffin, and in our experience protein epitopes are better preserved than mRNA in such samples. Since MMP-1 functions are tightly regulated by tissue inhibitors of metalloproteinases (TIMPS) in tissues undergoing inflammation [27]–[29], we used a polyclonal antibody that detects only free MMP-1 (pro-MMP-1 and mature activated MMP-1).

**Figure 1:**
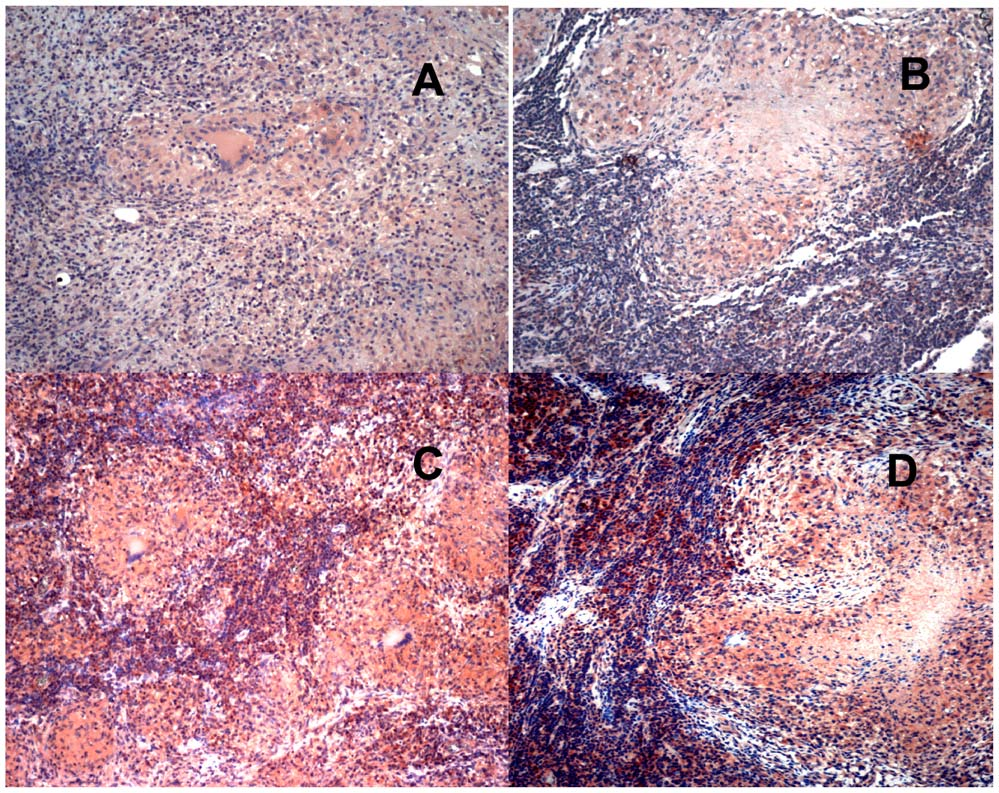
Carriers of the two-locus genotype *MCP-1 GG MMP-1 2G/2G* express more MMP-1 than carriers of any other two-locus genotypes tested. Immunohistochemical (IHC) analysis of MMP-1 expression in paraffin-embedded lymph-nodes from four representative Peruvian TB cases. Negative controls are shown in Figure S3. Representative lymph-nodes from each of the following two-locus genotypes are shown: A, *MCP-1 A- MMP-1 1G/-;* B, *MCP-1 A/- MMP-1 2g/2G*; C, *MCP-1 GG MMP-1 1G/-*; D, *MCP-1 GG MMP-1 2G/2G*. Images were acquired at 100× total magnification. Carriers of the two-locus genotype *MCP-1 GG MMP-1 2G/2G* showed larger extensions of tissue and more cells that stained dark red than tissue sections from carriers of any other combination of genotypes.

**Figure 2:**
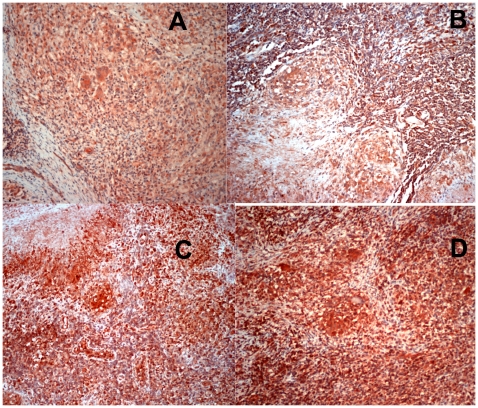
Carriers of the two-locus genotype *MCP-1 G/G MMP-1 2G/2G* express more MCP-1 than carriers of other two-locus genotypes tested. Immunohistochemical (IHC) analysis of MCP-1 expression in paraffin-embedded lymph-nodes from four representative Peruvian TB cases. Negative controls are shown in Figure S3. Representative lymph-node from each of the following two-locus genotypes is shown: A, *MCP-1 A- MMP-1 1G/-*; B, *MCP-1 A/- MMP-1 2g/2G*; C, *MCP-1 GG MMP-1 1G/-*; D, *MCP-1 GG MMP-1 2G/2G*. Images were acquired at 100× total magnification. Carriers of two-locus genotype *MCP-1 GG MMP-1 2G/2G* showed larger extensions of tissue and more cells that stained dark red than tissue sections from carriers of any other combination of genotypes.

All nodes tested showed mainly paucicellular and fibrotic old granulomas, and a great deal of tissue damage as reflected by the loss of normal lymph node histology (Figure 1 and 2). After examining the slides, two trained observers arrived at the consensus that carriers of the genotypes *MCP-1 GG* and *MMP-1 2G2G* have larger extensions of tissue containing cells expressing the highest amounts of MCP-1 and MMP-1 (dark red) than carriers of any other two-locus genotypes investigated (Kappa coefficient of agreement  = 0.67, Figure 1). Increased production of MCP-1 and MMP-1 might increase the chances of detecting positive cells, which could result in a perceived increased in the number of cells expressing these factors (a threshold effect). For this reason, we also examined whether differences in the proportion of cells expressing high levels of MCP-1 and MMP-1 could be detected between tissue samples from carriers of the relevant two-locus genotypes under study (Table 7). The proportion of cells expressing these two factors (number of positive cells divided by the total number of cells counted) was approximately 4- to 6-fold higher for MCP-1 and approximately 6- to 13-fold higher for MMP-1 in carriers of the two-locus genotype *MCP-1 GG MMP-1 2G/2G* than in carriers of any other relevant two-locus genotypes studied (Table 7). We found significant differences between carriers of the relevant two-locus genotypes studied in the mean number of cells stained positive for MCP-1 (ANOVA F = 232.68, *p* = 0.00001) and in the mean number of cells stained positive for MMP-1 (ANOVA F = 244.43, *p* = 0.00001). The Bonferroni pair-wise comparison indicates that the number of cells expressing MCP-1 was significantly greater in carriers of the two-locus genotype *MCP-1 GG MMP-1 2G/2G* than in carriers of two-locus genotypes *MCP-1 A/- MMP-1 1G/-* (*p<*0.001), *MCP-1 A/- MMP-1 2G/2G* (*p<*0.001), or *MCP-1 GG MMP-1 1G/-* (*p<*0.001). No other pair-wise comparisons were significant. Likewise, the number of cells expressing MMP-1 in carriers of the genotypes *MCP-1 GG MMP1 2G/2G* was significantly greater than in carriers of genotypes *MCP-1 A/- MMP-1 1G/-* (*p<*0.001), *MCP-1 A/- MMP-1 2G/2G* (*p<*0.001), or *MCP-1 GG MMP-1 1G/-* (*p<*0.001). The Bonferroni pair-wise comparison of carriers of genotypes *MCP-1 A /- MMP-1G/-* and *MCP-1 GG MMP-1 1G/-* also showed significant differences in the number of MMP-1 positive cells (*p* = 0.019), while other comparisons did not show significant differences. Thus, carriers of the two-locus *MCP-1 MMP1* genotype *GG 2G/2G* have the highest numbers of cells expressing both MCP-1 and MMP-1.

**Table 7:** Proportion of cells expressing high levels of MCP-1 and MMP-1 in lymph nodes from cases of tuberculosis carriers of -2518 *MCP-1* and -1607 *MMP-1* two-locus genotypes A.

Genotypes	Total No of cell countedmean ± SD	Total No of positive cellsmean ± SD (%)	Bonferroni LSD test B
***MCP-1 assessment***			
*A/- 1G/-*	151±19.3	10±5.1 (7)	*A/- 1G/-* versus *GG 2G/2G p*<0.001
*A/- 2G/2G*	184±30.5	11.3±7.5 (6)	*A/- 2G/2G* versus. *GG 2G/2G p*<0.001
*GG 1G/-*	187±25.8	23.6±3 (12.6)	*GG 1G/-* versus *GG 2G/2G p*<0.001
*GG 2G/2G*	206±9.5	98.8±14.4 (48)	
***MMP-1 assessment***			
*A/- 1G/-*	179±42.7	5.5±2 (3.2)	*A/- 1G/-* versus *GG 2G/2G p*<0.001
*A/- 2G/2G*	217.2±28.5	7.6±6 (3.3)	*A/- 1G/-* versus *GG 1G/- p* = 0.019
*GG 1G/-*	208.4±16.3	15.7±3 (7.5)	*A/- 2G/2G* versus *GG 2G/2G p*<0.001
*GG 2G/2G*	201.2±38.8	83.6±18.6 (42)	*GG 1G/-* versus *GG 2G/2G p*<0.001

A Immunohistochemical (IHC) staining of lymph node sections. We show the mean ± standard deviation (SD) of the number (No) of cells counted from four tuberculosis cases for each two-locus genotype. Procedures for the selection of lymph nodes, genotyping, IHC, and the counting of stained cells are explained in detail in Materials and Methods.

B We show comparisons that reach significant *p-*values in the Bonferroni least significant difference (LSD) pair-wise comparison test. These *p-*values were obtained after running one-way ANOVA tests. The means for the number of positive cells were significantly different between the different *MCP-1 MMP-1* two-locus genotype groups tested for MCP-1 (ANOVA F =  232.68, *p* **= **0.00001) and MMP-1 (ANOVA F** = **244.43, *p* **= **0.00001).

Cells producing large amounts of MMP-1 and MCP-1 were mainly located in areas surrounding old granulomas (Figures 1 and 2). Cells producing copious amounts of MCP-1 and MMP-1 (dark red) were also observed adjacent to or surrounding the necrotic areas (Figure S2). Monocytes produce MCP-1 and MMP-1 in response to *M. tuberculosis* antigens and their presence in *M. tuberculosis*-induced granulomas is a hallmark of TB infection (1, 28–30). Consistent with this, we observed that cells of the monocytic lineage were among the most important sources of both, MCP-1 and MMP-1 (Figure S2). A panel of appropriate negative controls is shown in Figure S3.

### Analysis of MCP-1 and MMP-1 Biological Interactions

**Figure 3:**
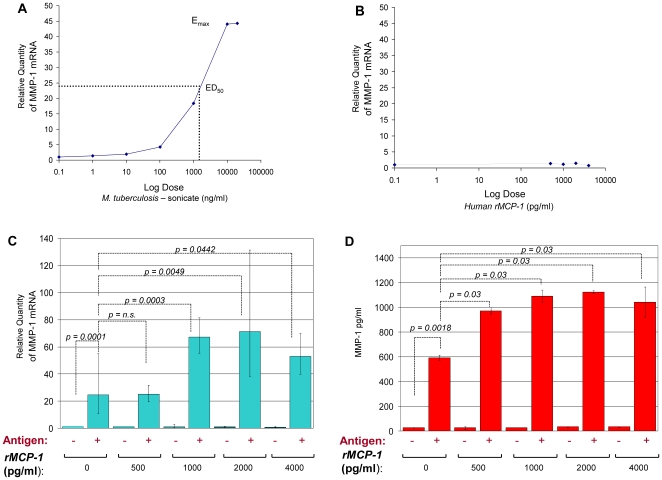
MCP-1 increases *MMP-1* expression inTHP-1 monocytic cells stimulated by *M. tuberculosis-sonicate antigens*. We measured the relative changes in *MMP-1* gene expression by real-time PCR. Data are presented as the fold change in gene expression normalized to the endogenous reference gene *PDHB* and relative to untreated controls (RQ values). A, *MMP-1* expression following 24 hours *in vitro* stimulation of THP-1 cells with the indicated amounts of *M. tuberculosis-*sonicate antigens. The effective dose 50 (ED50) of the *M. tuberculosis*-sonicate is indicated. B, *MMP-1* expression data following 24 hours *in vitro* stimulations of THP-1 cells with the indicated amounts of human recombinant MCP-1 (rMCP-1). Graph A and B show data from one of three experiments. C, *MMP-1* expression data for non-stimulated (−) and *M. tuberculosis*-sonicate antigen stimulated (+) THP-1 cells that were cultured for 24 hours with the indicated amounts of human rMCP-1. The results presented are from three independent experiments showing minimum and maximum RQ values. D, MMP-1 secretion levels for non-stimulated (−) and *M. tuberculosis*-sonicate antigen stimulated (+) THP-1 cells that were cultured for 48 hours in serum-free media with or without increasing amounts of human rMCP-1. Results presented are from three independent experiments and the bars indicate the standard deviation from the mean. The ED50 of *M. tuberculosis*-sonicate was used to stimulate the cells for the experiments shown in B, C, and D. The *p-*values from Wilcoxon-Mann-Whitney U-tests of comparisons of two independent variables are shown in C and D. Antigen  =  *M. tuberculosis*-sonicate 1000 ng/ml.

We developed an *in vitro* model to study whether MCP-1 interacts with MMP-1at the biological level. To test this in a meaningful way we used cells of the monocytic lineage, which are important constituents of granulomatous lesions in TB, and stimulated them with *M. tuberculosis*-sonicate antigens, human rMCP-1, or both. THP-1 monocytic cells were first stimulated with increasing amounts of *M. tuberculosis*-sonicate antigens or human rMCP-1 and expression of *MMP-1* was monitored by real-time PCR. Stimulation with *M. tuberculosis*-sonicate antigens induced *MMP-1* expression in a dose-dependent manner whereas human rMCP-1 alone did not (Figure 3A and 3B). As a positive control, human rMCP-1 alone induced the expression of CD11b by THP-1 cells, in a dose-dependent manner (data not shown). Stimulation of cells with *M. tuberculosis*-sonicate antigens alone significantly increased the expression of *MMP-1* (Z =  −3.85, *p* **= **0.0001, Figure 3C). Remarkably, addition of exogenous human rMCP-1 to THP-1 cells stimulated with *M. tuberculosis*-sonicate increased the expression of *MMP-1* mRNA in a dose-dependent fashion and to higher levels than in cells stimulated with antigen alone (Kruskal-Wallis *x* ^2^ = 27.13, *p* = 0.0001, Figure 3C). We observed significant differences in the levels of *MMP-1* mRNA when comparing cells stimulated with *M. tuberculosis* antigen alone versus cells stimulated with antigen plus 1000 pg/ml of rMCP-1 (Z = −3.62, *p* = 0.0003), 2000 pg/ml of rMCP-1 (Z = −2.817, *p* = 0.0049), or 4000 pg/ml of rMCP-1 (Z = −2.012, *p* = 0.0442) (Figure 3C). The levels of *MMP-1* mRNA expression were significantly greater in cells stimulated with *M. tuberculosis*-sonicate plus the various concentrations of rMCP-1 than in cells stimulated with *M. tuberculosis*-sonicate antigens alone, with the exception of cells stimulated with *M. tuberculosis*-sonicate antigens plus 500 pg/ml of rMCP-1 (Figure 3C). Addition of rMCP-1 alone at various concentrations did not induce *MMP-1* expression.

Accumulation of free MMP-1 protein (not neutralized by TIMPs or alpha-2-macroglobulins) was preserved in this *in vitro* system (Figure 3D) [27]. The data reported in Figure 3D were obtained using a functional assay that assessed levels of active protein in the culture media of stimulated cells (see Materials and Methods). Although it is evident that rMCP-1 induces a dose-dependent incremental accumulation of MMP-1 in culture media, testing for differences in the mean MMP-1 concentrations between variables was not significant using the Kruskal-Wallis test (Figure 3D). However, the accumulation of MMP-1 was greater in cells stimulated with *M. tuberculosis*-sonicate antigens than that in non-stimulated cells (Z = −3.127, *p* = 0.0018). Moreover, the accumulation of MMP-1 in culture media of cells stimulated with *M. tuberculosis*-sonicate antigens plus rMCP-1 was always significantly greater than in that culture media from cells stimulated with antigen alone. The Z and *p*-values obtained using the Wilcoxon-Mann-Whitney U-test to compare two independent samples were always significant and did not change in magnitude over all comparisons performed (Z = −2.966, *p* = 0.03, Figure 3D) because there were no statistically significant differences in the levels of MMP-1 accumulation in culture media of cells stimulated with antigen plus various doses of rMCP-1. Nevertheless, the results from these experiments support the idea that MCP-1 potentiates the induction of MMP-1 production following stimulation of cells of the monocytic lineage by *M. tuberculosis*-sonicate antigens. Non-stimulated control cells did not show significant MMP-1 secretion (Figures 3C and 3D). Hence, we concluded that MCP-1 potentiates *MMP-1* mRNA expression and MMP-1 secretion by cells of the monocytic lineage following stimulation by *M. tuberculosis*-sonicate antigens.

### Analysis of the Effects of *M. tuberculosis*-Sonicate Antigens and MCP-1 on the Activation of -1607 *MMP-1* Promoter Variants *1G* and *2G*

**Figure 4:**
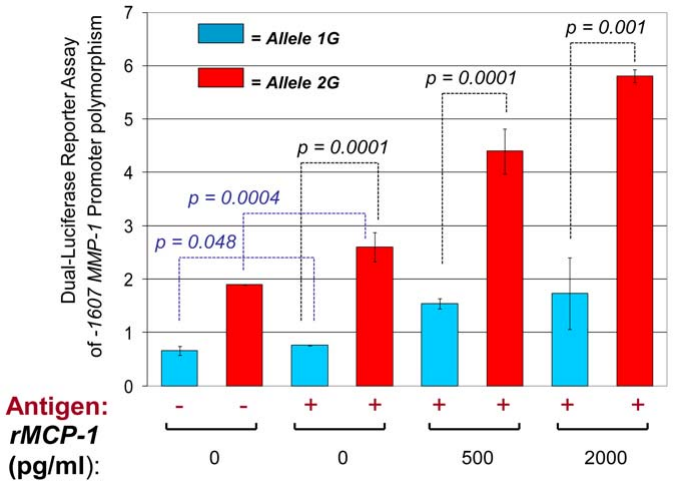
Luciferase activity in response to stimulation with *M. tuberculosis*-sonicate antigens and human rMCP-1 is greater in THP-1 cells transfected with the -1607 *MMP-1 2G* variant than in those transfected with the *MMP-1 1G* promoter variant. The activities of promoters carrying the -1607 alleles *1G* and *2G* were compared using a dual-luciferase assay system (see Materials and Methods for details). Cells were stimulated overnight with or without *M. tuberculosis*-sonicate antigens and with the indicated amounts of human recombinant MCP-1. The data are presented as the ratio of Firefly Luciferase signal to Renilla Luciferase (control) signal. The error bars represent the standard deviation of means obtained from four independent experiments. Blue bars indicate cells transfected with plasmids containing the -1607 *MMP-1 1G* variant and red bars show data from cells transfected with plasmids containing the *2G* variant. Addition of human rMCP-1 to cells stimulated with *M. tuberculosis*-sonicate antigens increased specific luciferase activity in a dose-dependent manner and to a level greater than that induced by *M. tuberculosis* antigens alone. The *p-*values from student t-tests are shown. In blue we show the *p-*values from comparisons of non-stimulated cells and cells stimulated with *M. tuberculosis*-sonicate antigens alone. Antigen  =  *M. tuberculosis*-sonicate 10 ng/ml.

Our *in vitro* model also allowed us to assess whether the -1607 *MMP-1* allele *2G* induces a higher promoter activity than the allele *1G* in cells of the monocytic lineage that are simulated with *M. tuberculosis*-sonicate antigens alone or in the presence of human rMCP-1. We used a dual luciferase assay to control for the efficiency of transfections [31]. The results of the luciferase assays were in agreement with those obtained from the *ex vivo* IHC analysis of lymph nodes and in our *in vitro* experiments outlined above. *M. tuberculosis*-antigen stimulated cells transfected with vector containing the -1607 *MMP-1* allele *1G* expressed higher luciferase activity than non-stimulated cells transfected with the same vector, but the difference was marginally significant (t = 2.48, *p* = 0.048) (Figure 4). In contrast, antigen stimulated cells transfected with vector containing the allele *2G* expressed significantly higher luciferase activity than non-stimulated cells transfected with the same vector (t = 6.99, *p* = 0.0004, Figure 4). Addition of human rMCP-1 to antigen stimulated cells increased the levels of luciferase activity in a dose-dependent manner beyond those observed in cells stimulated with antigen alone (Figure 4). The increments in luciferase activity produced by the addition of increasing amounts of human rMCP-1 to antigen stimulated cells were significant for the allele *1G* (ANOVA F = 10.23, *p* = 0.005) and highly significant for the allele *2G* (ANOVA F = 172.3, *p*<0.00001). Remarkably, cells transfected with vector containing the *MMP-1* allele *2G* expressed significantly higher levels of luciferase activity than cells transfected with vector containing the *MMP-1* allele *1G* when stimulated with *M. tuberculosis* antigen alone (t = 18.38, *p* = 0.0001), with antigen plus 500 pg/ml of human rMCP-1 (t = 13.32, *p* = 0.0001), or with antigen plus 2000 pg/ml human rMCP-1 (t = 11.99, *p* = 0.001) (Figure 4).

## Discussion

This study of BCG-vaccinated Mexicans and Peruvians reveals a significant and consistent joint effect of the -2518 *MCP-1* genotype *GG* and the -1607 *MMP-1* genotype *2G/2G*. The two-locus genotype *MCP-1 GG MMP-1 2G/2G* increased the likelihood of developing TB 3.59-fold in Mexicans and 3.9-fold in Peruvians. To build a conceptual frame upon which to understand the significance of our main finding we will first discuss our results from the univariate analysis of associations.

The current point estimates of independent associations of the susceptibility -2518 *MCP-1* genotype *GG* are significantly lower than those previously observed in non BCG-vaccinated Mexicans (OR = 5.4) and Koreans (OR = 6.9) [1]. The odd ratios (OR) obtained in the present study were 2.66 in Mexicans and 1.43 in Peruvians. In addition, we did not see a dose effect of the *MCP-1* susceptibility allele *G*, and consequently did not see an association of *MCP-1* genotype *AG* with disease in BCG-vaccinated Mexicans or Peruvians. Comparison between our current and previous data obtained in Mexican cases and controls suggests that BCG vaccination may have modified the effect of the -2518 *MCP-1* allele *G* and genotype *GG* on disease progression although other factors may have contributed. For example, differences in the virulence of circulating bacilli between the periods when we recruited the previous and the current Mexican samples. However, it should be noted that both Mexican samples are from the same geographical area, although they were obtained at slightly different periods of time. Moreover, the demographics and clinical features of Mexican cases and controls recruited for both studies were similar, with the only exception being the BCG vaccination status. We could not confirm this finding in Peruvians since it was not feasible to recruit unvaccinated cases or controls in Peru. Although it is likely that we would find non BCG-vaccinated individuals in remote villages in Peru, these villages are difficult to access. However, it is worth mentioning that clinicians have observed that BCG vaccination modifies the rate of progression from infection to active TB [16]. This finding highlights the need to pay special attention to the BCG vaccination status of cases and controls to avoid misclassification of the controls and bias when searching for genetic determinants of susceptibility to progression from infection to active tuberculosis.

It is interesting that the point estimates of *MCP-1* association with disease in Mexicans were greater in strength that those observed in Peruvians in the present study. Differences in the genomic structure and antigenic composition of the BCG sub-strains used for vaccination in Peruvians and Mexicans may in part explain these results [32], [33]. In Mexico, a BCG sub-strain derived from the BCG Copenhagen (Denmark) 1331 sub-strain has been in use since 1927 [34]. In Peru, the original BCG strain or the BCG sub-strain Moscow was used from 1924 to 1990 [34]–[36]. Since our youngest cases and controls from Peru were born before 1991, it is very likely that most of them were immunized with the original BCG or the BCG Moscow sub-strain.

In animal models, diverse BCG sub-strains exhibit varied protective efficacy against experimental infection with *M. tuberculosis* and induced qualitatively different immune responses [37]. Thus, certain sub-strains of BCG might induce a modest protective immunity that could slightly modify the effect of certain susceptibility genotypes. This might be the case with the BCG Mexico sub-strain. Alternatively, certain BCG sub-strains may induce an overwhelmingly intense pro-inflammatory recalled response to mycobacterial antigens that in turn may promote tissue damage independent of the effect of certain susceptibility genotypes. This might be the case for the original BCG or BCG Moscow used in Peru. It is worth mentioning the results of a clinical trial conducted in Mexican children to assess the expression profiles induced by different BCG sub-strains [38] that showed that the sub-strain Japan - which is structurally close to the Moscow sub-strain used in Peru - induces a more prominent recalled pro-inflammatory response than the BCG Copenhagen 1331 sub-strain used in Mexico [38]. The intense inflammatory response induced by certain BCG sub-strains may over-ride the protective effect of certain genotypes.

Other prominent factors may have also contributed to differences in the point estimates of *MCP-1* genotype *GG* association with disease in Mexicans and Peruvians, such as a more intense exposure to *M. tuberculosis* bacilli or the lower socioeconomic conditions in Peru. While Peruvians live in a moderate to high TB burdened country, the TB burden is lower in Mexico and Mexicans enjoy a relatively better socioeconomic status than Peruvians [15], [39], [40]. Poor living conditions may partially over-ride the independent effect of some loci, including the effect of genotypes that confer resistance to disease progression. In addition, a more pathogenic *M. tuberculosis* strain might be circulating in Peru. For example, in a study of *M. tuberculosis* samples from various Latin-American countries, 5.9% of the isolates from 185 TB cases belonged to the Beijing family [41]. Although there are no data on the incidence of this strain in Mexico, the same study reported that the incidence of this strain was markedly lower in samples from Argentinean TB cases (1% of 512 isolates), Brazilian TB cases (0.8% of 252 isolates), and Paraguayan TB cases (0.6% of 166 isolates), than in the Peruvian samples [41].

An association between the – 2518 *MCP-1* allele *G* and resistance to TB was observed in Ghanaians [42]. This association was attributed to the -362 *MCP-1* allele *C* inherited in linkage disequilibrium with the - 2518 *MCP-1* allele *G* [42]. The same study found no association of *MCP-1* with tuberculosis in a sample from Russia [42]. In a Chinese population from Hong Kong, no associations of *MCP-1* with tuberculosis was found [42], [43]. Notably, association of the *MCP-1* genotype *GG* with susceptibility to developing TB was found in a more ethnically homogeneous population of Han Chinese TB cases and controls [44]. Differences in study design may explain these disparate results, particularly with respect to the criteria for selection of controls, including the adjustment for BCG vaccination and nutritional status, the stringency of the methodology used for the correct ascertainment of exposure and “latent” infection status, and the utilization of methods to correct for genetic admixture, or in the criteria to detect and correct genotyping or sampling errors reflected in departure from Hardy-Weinberg equilibrium [1], [17], [20], [23], [45]–[47].

In this study we carefully established a cut-off at which we could increase the specificity of the PPD tests to detect latently infected controls among BCG-vaccinated individuals. To do this, we used the QuantiFERON-TB in-tube test, which discriminates between immunity induced by BCG vaccination and that induced by *M. tuberculosis* infection (see the Materials and Methods section) [48]. This test is highly sensitive and specific in detecting *M. tuberculosis* infected individuals [48]. Hence, all the controls in the present study had a PPD response >15 mm and were very likely “latently” infected with *M. tuberculosis* bacilli [16]–[20]. Since they were “latently” infected and might progress to disease, we ensured that they remained healthy for a period of at least two years after exposure [16], [17]. This provided a control population to identify genetic determinants of the progression from *M. tuberculosis* infection to active TB disease [1]. The criteria for the ascertainment of tuberculosis cases in the present study were very stringent and based on clinical features (signs and symptoms of TB), chest X-ray findings, and positive sputum-smear tests. However, these were not culture proven cases of *M. tuberculosis* infection and this might be seen as a source of bias since there is a remote possibility that they were infected with non-tuberculous mycobacterias (NTM) instead of *M. tuberculosis*. We think that this constitutes a minor limitation because the incidence of pulmonary TB caused by NTM is extremely low compare with that caused by *M. tuberculosis* [49], [50]. Moreover, NTM infection produces pulmonary disease when host immunity is impaired and/or in patients suffering from other chronic lung diseases [49], [50], which was not the case in our tuberculosis patients since we excluded individuals who were affected by immuno-deficiencies, receiving treatments that compromise immunity, or suffering from other lung diseases. In addition, if our cases were infected with NTM we would have needed extended periods of multidrug therapy to attain sterilization of the sputum (sputum negativity) [49], [50], and all our TB cases attained sputum negativity in the first 4 months of treatment. Moreover, recidivating disease is very common among cases of NTM-caused pulmonary disease [49], [50], and, we excluded TB cases suffering from recidivating TB. Hence, it is very unlikely that any of our cases were infected with NTM rather than *M. tuberculosis*. None of the studies reporting discrepant findings adopted our stringent criteria for the selection of controls.

The frequency of the susceptibility -2518 *MCP-1* allele *G* is markedly higher in Mexicans (0.45, Table 2) and Peruvians (0.64, Table 2) than in Caucasians (0.15 to 0.242) and non-Caucasian Africans (0.05 to 0. 175) [51]. Consequently, ours discrepant results may also reflect ethnic-specific characteristics resulting from unique environments and selective evolutionary pressures [52], as demonstrated for the *natural resistance-associated macrophage protein* (*NRAMP)-1* gene [52], [53]. Notably, we have experimentally observed that the -362 *MCP-1* polymorphism is not functional (Table S2) and do not think that the findings in Ghanaians are biologically relevant [42]. The contribution of the -1607 *MMP-1* genotype *2G/2G* to the expression of active pulmonary TB might also be population-specific since the frequency of the allele *2G* in Caucasians (0.433) and non-Caucasian Africans (0.375) is lower than in Mexicans (0.73) and Peruvians (0.71) (Table 2) [54].

Our results from the genetic analysis of joint effects support the notion that susceptibility to disease progression is a complex trait. Notably, 38% of Mexicans and 35% of Peruvians TB cases carry the two-locus susceptibility genotype *MCP-1 GG MMP-1 2G/2G*. However, 13% and 17% of latently infected healthy controls in Mexico and Peru also carry that susceptibility genotype and have not developed disease to date, indicating that other factors are involved in determining the fate of infection in these populations, including other genetic loci [45]. Our analysis of lymph nodes from TB cases and the results of our *in vitro* experiments support the hypothesis that carriers of the two-locus genotype *MCP-1 GG MMP-1 2G/2G* are at increased risk for progression to active TB because they express a unique phenotype characterized by high levels of both MCP-1 and free MMP-1. Our results from the dual-luciferase experimental model further support to this notion. Our *in vitro* data indicate that MCP-1 potentiates *M. tuberculosis*-antigens induction of *MMP-1* expression in cells of the monocytic lineage. Consequently, we observed numerous cells expressing large amounts of MCP-1 and MMP-1 adjacent to or surrounding necrotic areas in lymph nodes of cases with an ongoing inflammatory process. This support the notion that increased production of MMP-1 may destabilize the organization of new granulomas, thus contributing to the dissemination of infected cells and disease progression. Hence, we have brought together results from human population genetic and *in vitro* studies into a coherent hypothesis. Moreover, TB cases that are carriers of the two-locus genotype *MCP-1 GG MMP-1 2G/2G* may benefit from treatment to neutralize the deleterious effect of MMP-1.

In summary, our results indicate that BCG vaccination modifies the effect of the *MCP-1* susceptibility allele *G*. More studies are required to understand the mechanisms underlying this interesting effect. Most important, we show that the – 2518 *MCP-1* susceptibility genotype *GG* and the -1607 *MMP-1* genotype *2G/2G* jointly increase the likelihood of progression to active TB in a sub-group of BCG-vaccinated Mexicans and Peruvians. The strength and consistency of this association in both populations supports the notion that this joint effect is more important than the independent effects of either gene. Given that the two promoter polymorphisms are functional and both result in increased gene expression, we propose that increased levels of MCP-1, and consequently MMP-1, contribute to disease progression in BCG-vaccinated carriers of the two-locus genotype *MCP-1 GG MMP-1 2G/2G*. Increased MMP-1 availability and activity early in *M. tuberculosis* infection may destabilize granuloma formation, in particular the correct organization of new granulomas, promoting spread of the infection and progression to active TB. Animal models are needed to better understand how the two-locus genotype *MCP-1 GG MMP-1 2G/2G* may induce tissue damage in the lung during early stages of infection and to assess whether carriers of that two-locus genotype may benefit from treatment to neutralize the deleterious effect of excess production of MCP-1 and MMP-1.

## Materials and Methods

### Ethics Statement

All subjects provided informed consent, under protocols approved by the institutional review boards of the University of Texas Health Center at Tyler (the principal investigator spent 2 years as a faculty member in this institution), The Methodist Hospital (TMH) in Houston (Texas, USA), the Ministry of Health in Peru, and the Mexican National Institute of Medicine and Nutrition “Salvador Zubiran”.

### Study Subjects

We conducted case-control studies in Mexico and Peru. TB patients and controls were adults of Mestizo ethnicity aged 18 to 50 years, who were recruited in the cities of Mexico and in Lima and Callao in Peru, as part of the World Health Organization's DOTS community surveillance program for early detection of new TB cases [15]. Samples from Mexico were from the same geographical area as the cases and controls of our previous study [1]. From April 2006 through May 2007, 220 TB cases and 243 healthy controls were enrolled in Mexico, and from November 2005 through June 2009, 737 TB cases and 819 controls were enrolled in Peru. None of the potential participants declined to participate in the study. We could not obtain genotyping data for 27 TB cases from Mexico, and 36 TB cases and 23 controls from Peru. Thus, our sample size was 193 TB cases and 243 controls from Mexico, and 701 TB cases and 796 controls from Peru.

We applied most of the inclusion and exclusion criteria used in our previous study [1], with the only exception being BCG vaccination status. Thus, all cases and controls recruited in the present study were vaccinated with BCG at birth, had negative serologic tests for HIV infection, were of similar socioeconomic status within nationalities, and were unrelated to the third generation, as determined by a questionnaire. BCG vaccination status of cases and controls was confirmed by the presence of a scar in the left shoulder and vaccination records in the clinical charts. We assessed the nutritional status of our cases and controls using BMI as the main criterion [1], [55], [56]. The BMI for each subject was determined, based on self-reported weight, before disease in the case of TB patients, and height, measured by a nurse [1]. Individuals with a BMI <18.5 kg/m^2^ were considered to be malnourished [55], [56] and were excluded from the study because this condition compromises immunity and predisposes to disease progression [57].

All TB cases exhibited symptoms (weight loss >10 kg, cough, fever, night sweats for more than 1 month, or cervical or axillary lymphadenopathy) and chest radiographic findings consistent with pulmonary TB, a positive sputum acid-fast smear, and a history of close contact to a TB patient. Only new TB cases were included in the study. All cases developed disease less than 1 year after recorded exposure to an index case.

The controls were healthy latently infected individuals with a history of close contact to a sputum-smear positive TB patient for a period of more than one month. A tuberculin skin test was administered to all controls, using the Mantoux method to deliver 5 tuberculin units of purified protein derivative (Tubersol; Sanofi Pasteur Inc., Swifwater, PA) intradermally [18]–[20]. The diameter of induration was measured 48 hours after inoculation. We recruited controls that had a tuberculin reaction >15 mm of induration: by using this cut-off, we increased the specificity of the test [19], [20]. This strongly positive tuberculin reaction is much more likely to be caused by *M. tuberculosis* infection than by the BCG vaccination [16], [20].

To find the optimal PPD cut-off to distinguish infected from non-infected individuals, we performed a preliminary study in Peru involving 200 randomly selected and heavily exposed persons (household contacts of a sputum-smear positive TB case) who remained healthy for at least 2 years after exposure. Subjects were stratified into those with PPD >15 and those with PPD >10 mm but ≤15 mm. In each group, 100 randomly selected individuals were tested to determine whether they were infected with *M. tuberculosis* using the QuantiFERON (QFT)-TB Gold in-tube test (Cellestis, Inc., Valencia, CA). A positive QTF-TB test is an unequivocal sign of positive *M. tuberculosis* infection [48]. Among those with a PPD >15 mm, 91% were QFT-TB positive compared with only 48% of those with a PPD >10 mm but ≤15 mm. Hence, we decided to use a PPD >15 mm of induration as the cut-off for selection of controls.

To further ascertain that these control individuals were resistant to disease progression, we ensured that a period of at least 2 years since exposure to a TB patient had passed at the time of recruitment. The first 2 years is a critical period during which most of *M. tuberculosis* infected individuals who are susceptible to disease progression develop active TB [17]. All control individuals had negative sputum smears for acid-fast bacilli tests and normal chest radiographs. None of these healthy controls had received isoniazid, consistent with standard medical practice in Mexico and Peru. All potential controls whose tuberculin skin tests showed ≤15 mm of induration were excluded from the study.

### Blood Samples and Single Nucleotide Polymorphisms (SNPs) Analysis

Genomic DNA was isolated from blood cell pellets using DNA extraction kits (Qiagen, Valencia, CA). We genotyped the *-2518A>G* SNP in *MCP-1* (rs1024611), the -*1607_1608insG* variant in *MMP-1* (rs1799750), and 42 genomic control SNPs listed in Table S1. The rs1024611 and rs1799750 SNPs were genotyped in duplicate using the tetra-arms technique [58]. We did not find discrepancies in the typing results for *MCP-1* and *MMP-1*. We will make primer sequences available and procedures available upon request. To confirm our results for the *MCP-1* and *MMP-1* gene analysis, we sequenced 10 randomly selected Mexican and Peruvian cases and controls. Previously published genotyping results of genomic controls for Mexicans were applied to our sample of Mexicans in the present study [1]. The 42 SNPs used as genomic controls in Peruvians were genotyped using an Illumina platform. Given that Africans are the founders of all human races and that the -2518 *MCP-1* allele *A* and the -1607 *MMP-1* allele *1G* are the most frequent in this population [51], [54], we used them as reference alleles.

### *Ex Vivo* Analysis of Lymph-Nodes and *In Vitro* Studies

For immunohistochemistry (IHC), 35 axillary or cervical lymph nodes fixed in paraformaldehyde and embedded in paraffin were obtained from archived samples at the Hospital Maria Auxiliadora in Lima, Peru. All of these were from TB patients that met the inclusion/exclusion criteria described above. We then selected 16 TB cases that were carriers of the genotype combinations under study and had a lymph node inflammatory process of no more than 15 days of evolution. The lymph nodes analyzed were all from males with no significant differences in age, either within or between groups. We extracted genomic DNA from four core sections (80 µm thick) per sample, using the Recoverall Total Nucleic Acid Isolation kit (Applied Biosystems, Foster City, CA) and deparaffination and extraction protocols provided with the kit. The genomic DNA was genotyped as described above. For IHC we used heat-induced epitope retrieval in citrate buffer (Thermo/Fisher Scientific Inc., Waltham, MA), anti-human MMP-1/Collagenase-1 rabbit purified total polyclonal IgG antibody Ab-6 (Thermo/Fisher Scientific Inc) or anti-human MCP-1/CCL-2 mouse IgG2B monoclonal antibody from clone 23002 (R&D Systems, Minneapolis, MN), and the biotin-free HRP-polymer AEC-based detection system (UltraVision LP Detection System, Lab Vision Products, Fremont, CA). We immunostained 5-µm thick sections in duplicate for each case, using contrast Blue for counterstaining (KPL Inc., Gaithersburg, ME). Negative controls were sections incubated with irrelevant normal rabbit IgG (KPL Inc., Gaithersburg, ME) or irrelevant normal mouse IgG2B antibody (R&D Systems). We used 1X Automation buffer pH 7.5 (Biomeda Inc., Albuquerque, NM) in all wash steps. SuperMount permanent aqueous mounting media (BioGenex, San Ramon, CA) was used for mounting immunostained tissue sections. Immunostained tissue sections were first assessed at 100× or 200× total magnification and 0.8 numerical aperture of the objective lenses. The tissue sections were assessed by two trained observers who were blind to the clinical and genotyping information. Image acquisition was performed using a computerized analysis system comprising: a BX41 microscope with a U-TVIX-2 and a U-CMAD3 tube and adapter attached for on-screen viewing, a C3040 4.1 megapixel digital camera, and Magnafire-SP software (Olympus America Inc.). The number of cells expressing high levels of MCP-1 and MMP-1 was assessed at 400× magnification with the aid of a 10×10 mm ocular grid with 100 individual squares of 1 mm square (Microscope World, Carlsbad, CA). Five randomly selected fields per slide (one slide per individual with duplicate samples) were evaluated.

### Culture Conditions, RT-PCR and Dual-Luciferase Assay

For the assessment of *MMP-1* expression we used the 7500 Fast Real-Time PCR System. We used assay-on-demand primers for *MMP-1* (ID Hs00233958), CD11b (Hs00167304), and the house keeping gene *PDHB* (*Pyruvate dehydrogenase beta*, ID Hs00168650) (Applied Biosystems). To calculate the relative quantity (RQ) of MMP-1, we used the 2 ^-ΔΔCT^ method implemented in the software [59]. The data are presented as the fold change in gene expression normalized to the endogenous reference gene PDHB and relative to the untreated controls (RQ values). THP-1 cells (1×10^6^ cells/ml) were stimulated with the indicated amounts of *M. tuberculosis*-lysate obtained after sonication (*M. tuberculosis*-sonicate) as described previously [1], or the indicated amounts of human rMCP-1 (R&D Systems), or both. Cells were cultured for 24 and 48 hours in complete RPMI (10% FCS) or serum-free media (Macrophage SFM medium, Invitrogen, Carlsbad, CA) as indicated in the figure legends. Cells were then harvested and total RNA was extracted using TRIzol (Invitrogen). Complementary DNA (cDNA) was obtained from 3 µg of total RNA using the High Capacity cDNA Reverse Transcription Kit (Applied Biosystems). Approximately 100 ng cDNA was used to determine the expression levels of the housekeeping gene *PDHB* and *MMP-1*. Supernatants from the same cultures were harvested and kept at −20°C until analyzed. MMP-1 activity in the supernatants was tested using Fluorokine E, Human Active MMP-1 kits (R&D Systems) and the Multi-mode Microplate Reader Synergy 2 (BioTek Instruments Inc., Winooski, VT). This assay uses a monoclonal antibody to capture free MMP-1 and assesses its levels by measuring the amount of cleaved collagen peptide by monitoring fluorescence. Since the antibody captures only free MMP-1 and the assay is a functional assay, detection of MMP-1 that is complexed and neutralized by alpha-2-macroglobulin or TIMPs is excluded.

We compared the activity of promoters carrying the -1607 *MMP-1* alleles *1G* and *2G* using the dual-luciferase assay system and Glomax 96-microplate luminometer (Promega, Madison, WI). The *MMP-1* promoter region spanning positions −1350 to −1766 was cloned into the Firefly Luciferase vector pGL4.10[*luc2*] (Promega), expanded in DH5α *E. coli* cells (Invitrogen) and purified using an endotoxin free plasmid purification system (Qiagen). Alleles containing promoter fragments were obtained by PCR from genomic DNA obtained from -1607 *MMP-1* homozygous *1G/1G* and *2G/2G*. THP-1 cells were co-transfected with pGL4.10[*luc2*] vectors containing the allele *1G* or the allele *2G* and control pGL4 Renilla Luciferase vector pGL4.73[*hRluc/SV40*] (Promega) in a 50∶1 ratio using Effectene transfection reagents from Qiagen. Cells were cultured overnight in complete RPMI (10% FCS) and then washed three times in RPMI, re-suspended in complete RPMI, and stimulated overnight with or without *M. tuberculosis*-sonicate antigens and human rMCP-1. Protocols provided by the dual-luciferase assay system were followed for transfection of the cells, preparation of cell lysates, and measurements of the dual-luciferase signals.

### Statistical Analysis

Statistical analysis was performed using Intercooled STATA10 software (Stata Corporation, College Station, TX). Based on preliminary data for frequencies of alleles observed at the two loci in both populations, we calculated that our sample size would provide 90% power to detect an OR of 1.5 with a two-sided α of 0.025. Sample size calculations were performed as explained elsewhere [1]. Hardy-Weinberg equilibrium was calculated using chi-square (*x^2^*) tests for n(n+1)/2 degrees of freedom, where n is the number of alleles in the polymorphism tested [1], [47]. Expected genotype proportions were obtained using allele frequencies observed in the controls and the binomial equation [47].

#### Univariate analysis of categorical data

Association of alleles with disease was analyzed using 2×2 contingency tables, and two-sided *x^2^* tests [60]. The *x^2^* values were corrected for population stratification, primarily to control for differences in the level of admixture between cases and controls, by dividing the *x^2^* values by an estimated value of λ [23]. We used our previously calculated λ values for the Mexican sample [1]. For Peruvians λ was calculated as the mean of *x^2^* from comparison of the allele frequencies of 42 SNPs located across the genome in a randomly selected sample of 96 cases and 96 controls. SNPs selected as genomic controls were not in LD (Table S1) [23], [61]. The *x^2^*-values obtained from the analysis of allele associations with disease were divided by λ and the *p*-values calculated based on the adjusted *x^2^*-values. The resulting *p-*values were further adjusted according to the number of comparisons, using the Bonferroni correction (*p-values). We used the method of Reich and Goldstein to control for genetic admixture because this method provides a precise coefficient to correct for stratification while testing for associations [23]. This practical approach is unmatched by other proposed methods [23]. For example, methods that rely on principal component analysis to deal with stratification might not be practical since they impose the stratified analysis of sub-groups exhibiting similar characteristics, increasing dimensionality and the sample size required [62].

We used multiple 2×2 tables, with genotypes arranged in an ordinal scale, and *x^2^* Mantel-Haenszel statistics to test each of the three genotypes produced by the diallelic locus for association with disease, primarily to obtain valid estimates for association for each stratum, and to assess dose effects and homogeneity of the odd ratios (OR) across strata [46]. To test for homogeneity of OR we used the Breslow and Day's test provided in the SATA10 output. Before routinely collapsing information across strata we considered it important to obtain estimates of association for each stratum using *x^2^* Mantel-Haenszel statistics to make sure that they could be meaningfully pooled [46].

#### Multivariate analysis of categorical data

To test main and joint (interaction) effects, we used multiple logistic regression analysis. Based on the results from univariate analysis of genotypes we collapsed *MCP-1* and *MMP-1* genotypes that were not found to be associated with disease and ran a hypothesis driven analysis of joint effects. We declared a significant interaction effect if the observed joint OR was greater than the expected OR, and the Z statistics and *p* values were significant [24], [25]. We performed Pearson and Hosmer-Lemeshow goodness-of-fit *x* ^2^ tests to determine whether the observed models differed from the predicted [25]. The desirable outcome of non-significance (*p*>0.1) means that the predicted model does not differ from the observed [25]. The ORs for the additive model were obtained using STATA10 output and the following formula: (OR*_GG_* + OR*_2G/2G_*) – 1; were OR*_GG_* is the adjusted OR for the *MCP-1* genotype *GG* in the absence of *MMP-1* genotype *2G/2G* and OR*_2G/2G_* is the adjusted OR for the *MMP-1* genotype *2G/2G* in the absence of *MCP-1* genotype *GG* [24].

#### Other statistic methods

To analyze IHC results we used the Kappa statistic and a modified Rietveld and van Houd scale [63]. According to this scale the interpretation of Kappa coefficients is as follows: <0 =  Less than chance agreement, 0.01–0.2 =  Slight agreement, 0.21–0.40 =  Fair agreement, 0.41–0.60 =  Moderate agreement, 0.61–0.8 =  Substantial agreement, 0.81–0.99 =  Almost perfect agreement [63]. We performed one-way analysis of variance (ANOVA) to determine whether differences in the means number of positive cells between groups were significant [64]. Twenty observations for each genotype group (4 patients per group ×5 randomly selected fields per patient) were tested. ANOVA tests were followed by Bonferroni least significant difference pair-wise comparisons [64]. For the analysis of some *in vitro* experiments non-parametric Kruskal-Wallis or one-way ANOVA were used to compare mean values between groups, and Wilcoxon-Mann-Whitney U-test or student t-tests for independent variables were used to compare two groups mean values [64]. Before running ANOVA we confirmed normal distribution of the data and homocedasticity using Shapiro-Wilk and Barlett's tests, respectively [64]. If these assumptions were violated non-parametric tests were run.

## Supporting Information

### Supplemental Material

Figure S1 shows the BCG-modifier effect in Mexicans comparing the strength of OR and CI in the pool and in samples used in the previous and present studies. Figure S2 shows IHC of lymph nodes from TB cases in which cells expressing MCP-1 and MMP-1 producing surround necrotic areas. Figure S3 shows typical examples of negative controls results from IHC analysis. Table S1 lists SNPs typed as genomic controls, SNP name, chromosome location and position, allele characteristics, call rates (CR) for each SNP in cases and controls, genotype frequencies, Hardy-Weinberg equilibrium assessment (HWE), minor allele frequencies (MAF), and chi-square values resulting from comparison of allele frequencies. Table S2 shows results of the assessment of the effect of the *-362 G>C MCP-1* SNP (rs2857656) on *MCP-1* promoter activity.

Figure S1 BCG vaccination modifies the effect of the -2518 *MCP-1* susceptibility allele *G* and genotypes. Odd ratios (OR) and 95% confidence intervals (CI) are shown as measurements of the strength of associations between the -2518 *MCP-1 A* to *G* transition and progression from infection to active TB. Diamonds represent the OR and error bars represent the CI. An OR of 1 indicates no association. The further the value of the OR is from 1, in a negative or positive direction, the stronger the association. In the upper part of the figure, the pool of Mexican TB cases and the latently infected PPD+ controls from this and our previous study (1) is shown. The pool consists of 628 TB cases (61 homozygous *AA*, 245 heterozygous, and 322 homozygous *GG*) and 577 latently infected healthy PPD+ controls (128 homozygous *AA*, 288 heterozygous, and 161 homozygous *GG*). In the lower part of the figure the strata of non-BCG (left) vaccinated cases and controls from our previous study (1) and BCG (right) vaccinated cases and controls from the present study (Table 3) are shown. We used *x^2^* Mantel-Haenszel statistics to test for genotype association with disease progression.
(0.01 MB PDF)

Figure S2 Cells producing MMP-1 and MCP-1 are also located adjacent to necrotic areas. Immunohistochemical (IHC) analysis of MMP-1 and MCP-1 expression in paraffin-embedded lymph-nodes from Peruvian TB cases carriers of the two- locus genotype *MCP-1 GG MMP-1 2G/2G*. Arrows indicate necrotic areas surrounded by cells expressing large amounts of MMP-1 (A) and MCP-1 (B) in dark red. There are many cells with pycnotic (condensed) nuclei in A and B. Images were acquired at 200× total magnification.
(0.21 MB PDF)

Figure S3 Negative controls. Immunohistochemical (IHC) analysis of MMP-1 and MCP-1 expression in paraffin-embedded lymph-nodes from Peruvian TB cases presented in Figure 1 and 2. In A, negative control incubated with normal rabbit IgG. In B, negative control incubated with normal mouse IgG2B. Images were acquired at 100× total magnification.
(0.45 MB PDF)

Table S1 Genomic Controls. ^A^ These SNPs were genotyped as genomic controls. All SNPs selected are not in linkage disequilibrium and should segregate independently from each other. To control for errors in the estimation of chi-square (*x^2^*) values and HWE *p*-values all the SNPs selected have Minor Allele frequencies (MAF) >0.1 (high MAF). This ensured that we will have at least 10 out of 100 carriers of the rare allele and at least 1 homozygous for the rare allele in the dataset. We list SNP names, chromosome location and position, allele characteristics, call rates (CR) for each SNP in cases and controls, genotype frequencies (in black for controls and in red for cases), Hardy-Weinberg equilibrium assessment (HWE), minor allele frequencies (MAF), and chi-square (*x^2^*) values resulting from comparison of allele frequencies. ^B^ We included in the analysis those SNPs with call rates (CR) >95% to avoid errors in the estimation of *x^2^* values. We did not need to re-cluster any of the loci tested to obtained call rate values presented in the table. We reanalyzed five loci using Tetra-Arms technique and the concordance rate was 100%. Thus, genotyping results obtained with the Illunina platform are highly reproducible. ^C^ Hardy-Weinberg equilibrium (HWE) test was done using the BeadStudio 3.0 Genotyping Module (GT) software from Illumina. The Null hypothesis for the HWE tested is: the distribution of genotypes is not in Hardy-Weinberg equilibrium. All loci tested in the control population were in HWE. ^D^ A chi-square (*x^2^*) value >3.8 is significant at *p*<0.05, and a *x^2^* value >6.6 is significant at *p*<0.01 with 1 degree of freedom. A value of lambda was obtained as follows: λ = Σ *x^2^*/number of SNPs  = 51.723/42 = 1.2315.
(0.10 MB DOC)

Table S2 Luciferase activity in response to PMA and *M. tuberculosis*-sonicate antigens stimulation of THP-1 cells transfected with vectors containing the -362 *MCP-1* alleles *G* or *C*. ^A^ The promoter region spanning positions -537 to position -156 of *MCP-1* gene containing the -362 *MCP-1* alleles *G* and *C* were amplified from - 362 *MCP-1* homozygous *GG* or *CC* and cloned into the Firely Luciferase vector pGL4.10[*luc2*] (Promega Corporation, Madison, WI), expanded in DH5α *E. coli* cells (Invitrogen, Carlsbad, CA) and purified using an endotoxin free plasmid purification system (Qiagen, Valencia, CA). ^B^ THP-1 cells were co-transfected with pGL4.10[*luc2*] vectors containing the -362 *MCP-1* allele *G* or the allele *C* and control pGL4 Renilla Luciferase vector pGL4.73[*hRluc/SV40*] (Promega Corporation, Madison, WI) in a 50∶1 ratio using Effectene transfection reagents from Qiagen (Valencia, CA). Cells were cultured overnight in complete RPMI (10% FCS) and then washed three times in RPMI, re-suspended in complete RPMI, and stimulated overnight with or without PMA or *M. tuberculosis*-sonicate antigens. Protocols provided by the dual-luciferase assay system were followed for transfection of cells, preparation of cell lysates, and measurement of dual-luciferase signals. The data are presented as the ratio of Firefly Luciferase and Renilla Luciferase (control) signal. The standard deviation of means obtained from three independent experiments. ^C^ We run student t-tests using STATA10 to determine whether the alleles induce statistically different expression levels of luciferase in THP-1 cells. Vectors containing the -362 *MCP-1* alleles are available upon request.
(0.03 MB DOC)
