# Supplementary figures and images for: Joint Effect of MCP-1 Genotype GG and MMP-1 Genotype 2G/2G Increases the Likelihood of Developing Pulmonary Tuberculosis in BCG-Vaccinated Individuals
Source: PLoS One. 2010 Jan 25;5(1):e8881. doi: 10.1371/journal.pone.0008881 (PMC2810343; doi:10.1371/journal.pone.0008881)

### Total Mexican sample

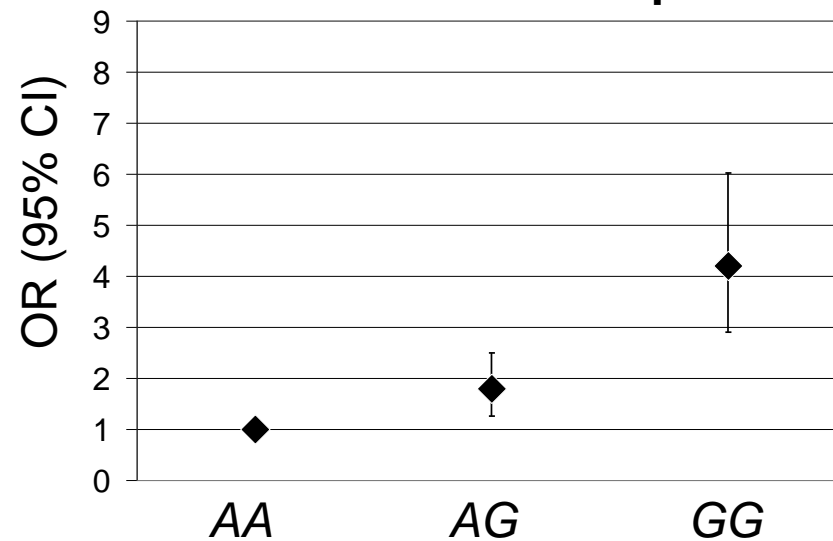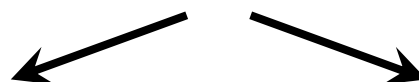

### Non-BCG vaccinated

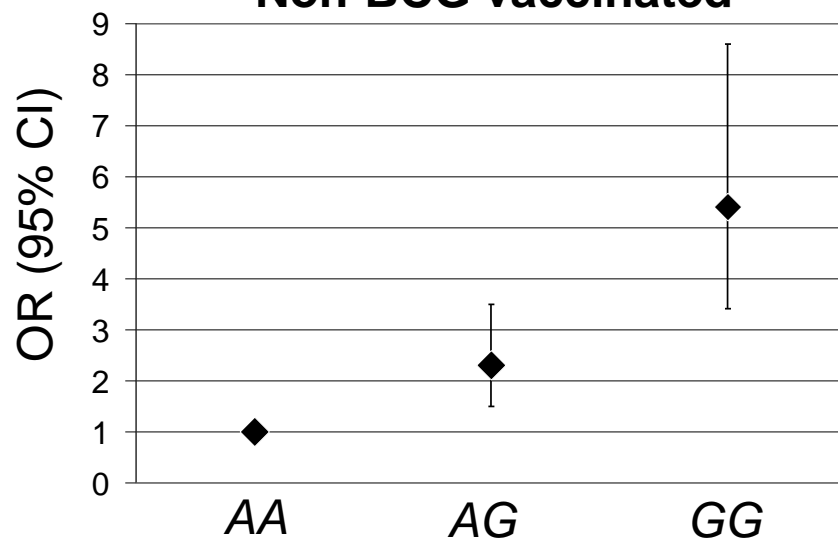

### BCG vaccinated

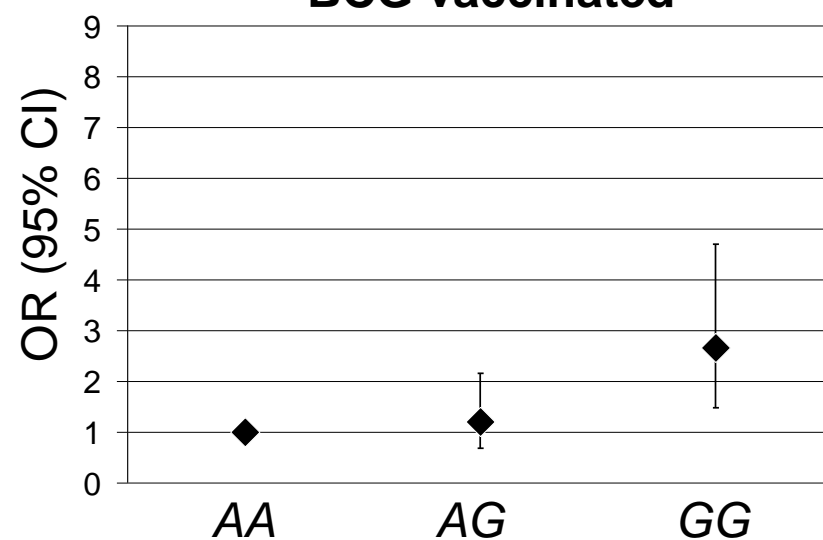

Supplement: Figure S1 — BCG vaccination modifies the effect of the -2518 MCP-1 susceptibility allele G and genotypes. Odd ratios (OR) and 95% confidence intervals (CI) are shown as measurements of the strength of associations between the -2518 MCP-1 A to G transition and progression from infection to active TB. Diamonds represent the OR and error bars represent the CI. An OR of 1 indicates no association. The further the value of the OR is from 1, in a negative or positive direction, the stronger the association. In the upper part of the figure, the pool of Mexican TB cases and the latently infected PPD+ controls from this and our previous study (1) is shown. The pool consists of 628 TB cases (61 homozygous AA, 245 heterozygous, and 322 homozygous GG) and 577 latently infected healthy PPD+ controls (128 homozygous AA, 288 heterozygous, and 161 homozygous GG). In the lower part of the figure the strata of non-BCG (left) vaccinated cases and controls from our previous study (1) and BCG (right) vaccinated cases and controls from the present study (Table 3) are shown. We used x2 Mantel-Haenszel statistics to test for genotype association with disease progression. (0.01 MB PDF) [file pone.0008881.s001.pdf]

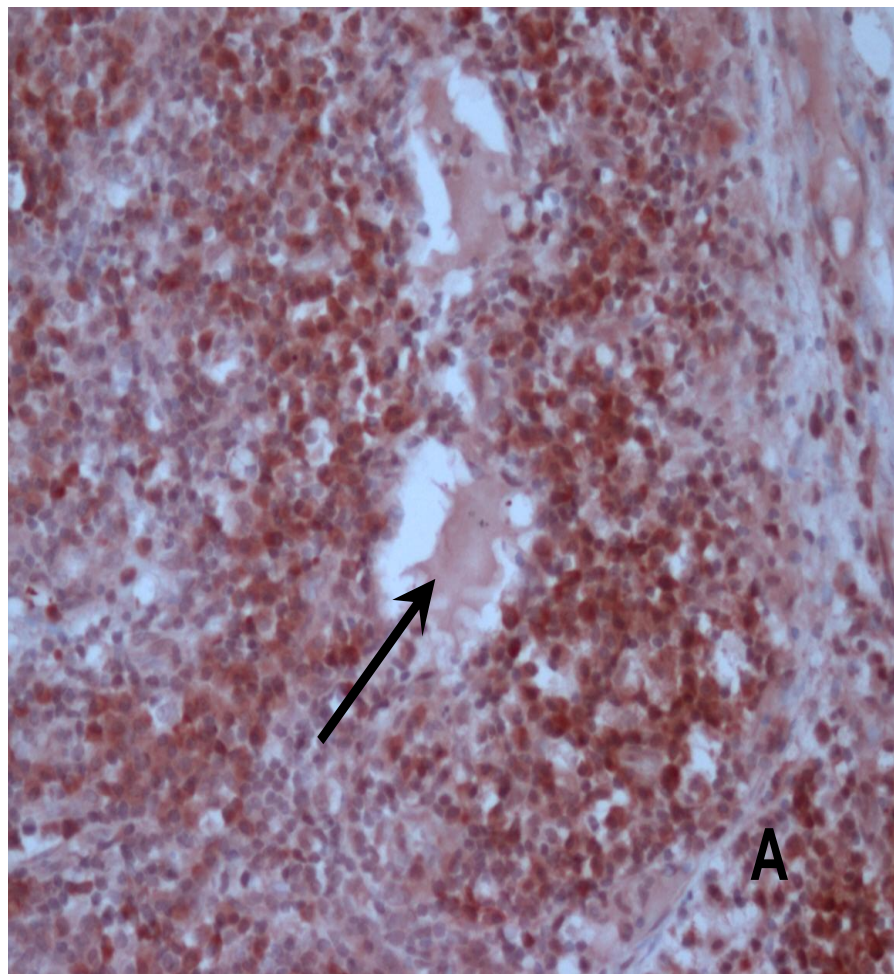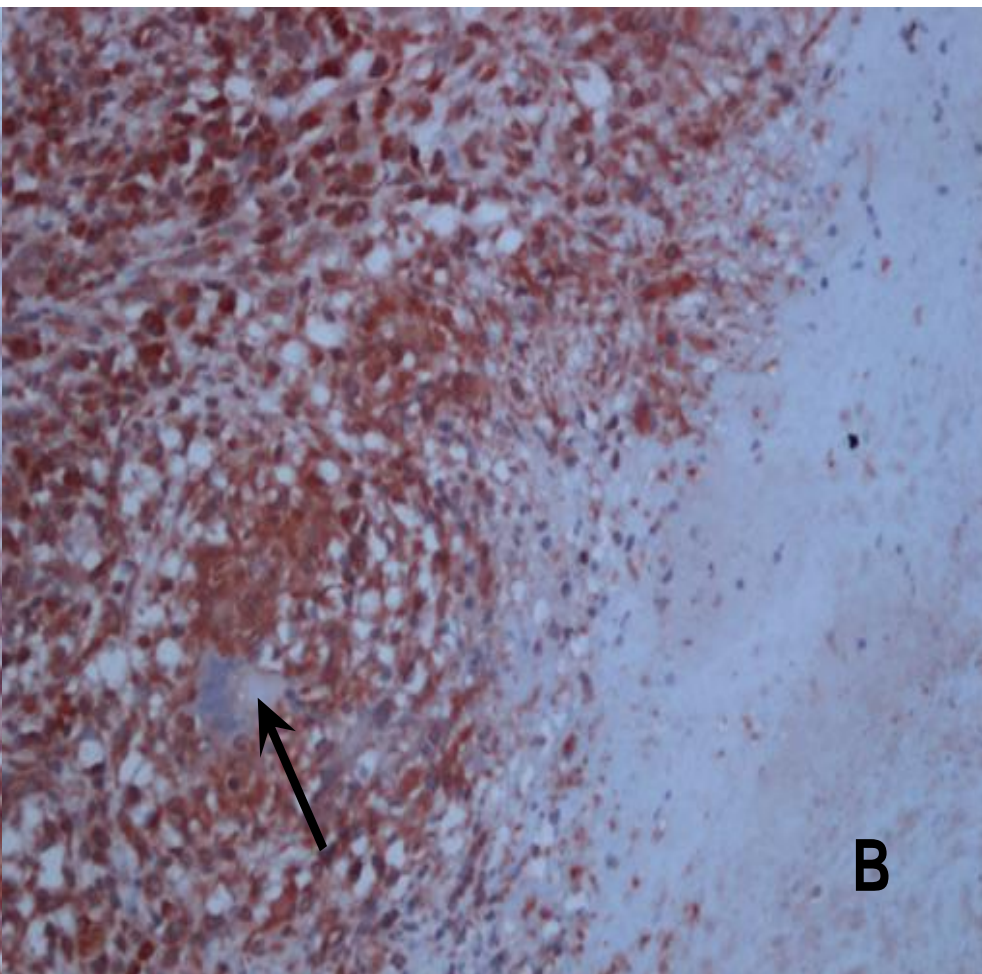

Supplement: Figure S2 — Cells producing MMP-1 and MCP-1 are also located adjacent to necrotic areas. Immunohistochemical (IHC) analysis of MMP-1 and MCP-1 expression in paraffin-embedded lymph-nodes from Peruvian TB cases carriers of the two- locus genotype MCP-1 GG MMP-1 2G/2G. Arrows indicate necrotic areas surrounded by cells expressing large amounts of MMP-1 (A) and MCP-1 (B) in dark red. There are many cells with pycnotic (condensed) nuclei in A and B. Images were acquired at 200× total magnification. (0.21 MB PDF) [file pone.0008881.s002.pdf]

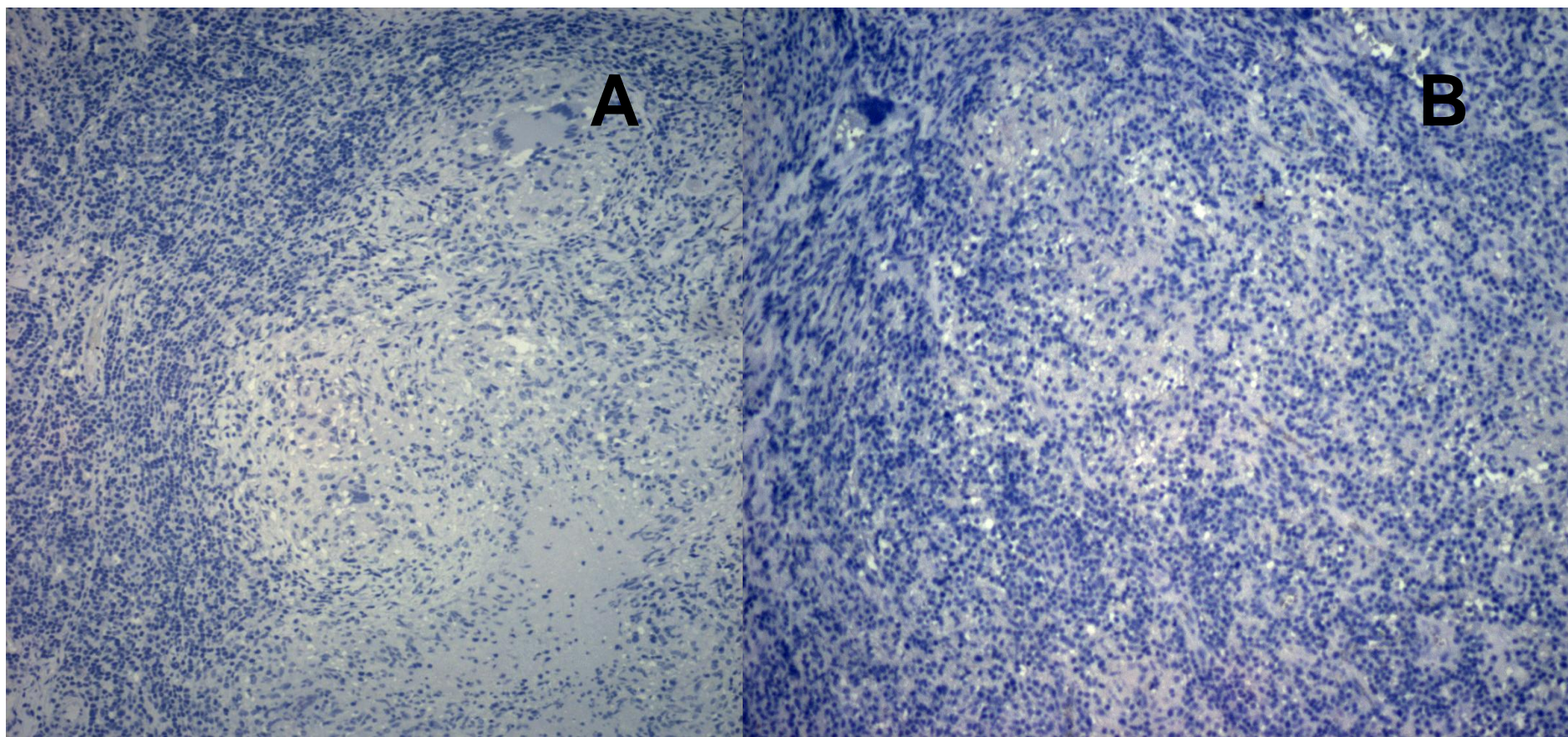

Supplement: Figure S3 — Negative controls. Immunohistochemical (IHC) analysis of MMP-1 and MCP-1 expression in paraffin-embedded lymph-nodes from Peruvian TB cases presented in Figure 1 and 2. In A, negative control incubated with normal rabbit IgG. In B, negative control incubated with normal mouse IgG2B. Images were acquired at 100× total magnification. (0.45 MB PDF) [file pone.0008881.s003.pdf]
