# Supplementary material for: Joint Effect of MCP-1 Genotype GG and MMP-1 Genotype 2G/2G Increases the Likelihood of Developing Pulmonary Tuberculosis in BCG-Vaccinated Individuals
Source: PLoS One. 2010 Jan 25;5(1):e8881. doi: 10.1371/journal.pone.0008881 (PMC2810343; doi:10.1371/journal.pone.0008881)
Supplement: Table S1 — Genomic Controls. A These SNPs were genotyped as genomic controls. All SNPs selected are not in linkage disequilibrium and should segregate independently from each other. To control for errors in the estimation of chi-square (x2) values and HWE p-values all the SNPs selected have Minor Allele frequencies (MAF) >0.1 (high MAF). This ensured that we will have at least 10 out of 100 carriers of the rare allele and at least 1 homozygous for the rare allele in the dataset. We list SNP names, chromosome location and position, allele characteristics, call rates (CR) for each SNP in cases and controls, genotype frequencies (in black for controls and in red for cases), Hardy-Weinberg equilibrium assessment (HWE), minor allele frequencies (MAF), and chi-square (x2) values resulting from comparison of allele frequencies. B We included in the analysis those SNPs with call rates (CR) >95% to avoid errors in the estimation of x2 values. We did not need to re-cluster any of the loci tested to obtained call rate values presented in the table. We reanalyzed five loci using Tetra-Arms technique and the concordance rate was 100%. Thus, genotyping results obtained with the Illunina platform are highly reproducible. C Hardy-Weinberg equilibrium (HWE) test was done using the BeadStudio 3.0 Genotyping Module (GT) software from Illumina. The Null hypothesis for the HWE tested is: the distribution of genotypes is not in Hardy-Weinberg equilibrium. All loci tested in the control population were in HWE. D A chi-square (x2) value >3.8 is significant at p<0.05, and a x2 value >6.6 is significant at p<0.01 with 1 degree of freedom. A value of lambda was obtained as follows: λ = Σ x2/number of SNPs = 51.723/42 = 1.2315. (0.10 MB DOC) [file pone.0008881.s004.doc]

| **SNP Name ^A^** | **Chr** | **Position** | **SNP** | **CR**  **Controls^B^**  **Cases** | **AA Freq**  **Controls**  **Cases** | **AB Freq**  **Controls**  **Cases** | **BB Freq**  **Controls**  **Cases** | **HWE (p-value)^C^**  **Controls**  **Cases** | **MAF**  **Controls**  **Cases** | **Allele Freq**  **Chi-square^D^** |
| --- | --- | --- | --- | --- | --- | --- | --- | --- | --- | --- |
| **rs10910518** | **1** | **230658690** | **[A/C]** | **1**  **1** | **(50) 0.5208**  **(42) 0.4375** | **(39) 0.4063**  **(42) 0.4375** | **(7) 0.0729**  **(12) 0.125** | **0.8695**  **0.7619** | **0.276**  **0.3438** | **2.06** |
| **rs4110479** | **1** | **232415613** | **[T/C]** | **1**  **1** | **(39) 0.4063**  **(33) 0.3438** | **(41) 0.4271**  **(42) 0.4375** | **(16) 0.1667**  **(21) 0.2188** | **0.3482**  **0.2665** | **0.3802**  **0.4375** | **1.3** |
| **rs12613171** | **2** | **96917474** | **[A/G]** | **1**  **1** | **(31) 0.3229**  **(39) 0.4063** | **(47) 0.4896**  **(45) 0.4688** | **(18) 0.1875**  **(12) 0.125** | **0.9797**  **0.8569** | **0.4323**  **0.3594** | **2.13** |
| **rs11682222** | **2** | **106167688** | **[T/C]** | **1**  **1** | **(14) 0.1458**  **(18) 0.1875** | **(43) 0.4479**  **(52) 0.5417** | **(39) 0.4063**  **(26) 0.2708** | **0.6966**  **0.3633** | **0.3698**  **0.4583** | **3.1** |
| **rs9838755** | **3** | **107007800** | **[A/G]** | **1**  **0.9896** | **(19) 0.1979**  **(16) 0.1684** | **(45) 0.4688**  **(42) 0.4421** | **(32) 0.3333**  **(37) 0.3895** | **0.6528**  **0.4817** | **0.4323**  **0.3895** | **0.26** |
| **rs6771904** | **3** | **152630421** | **[T/C]** | **1**  **1** | **(9) 0.0938**  **(15) 0.1563** | **(49) 0.5104**  **(42) 0.4421** | **(38) 0.3958**  **(37) 0.3895** | **0.2174**  **0.1291** | **0.349**  **0.349** | **0** |
| **rs7695134** | **4** | **62387447** | **[A/T]** | **1**  **1** | **(7) 0.0729**  **(3) 0.0313** | **(35) 0.3646**  **(30) 0.3125** | **(54) 0.5625**  **(63) 0.6563** | **0.6821**  **0.7976** | **0.2552**  **0.1875** | **2.55** |
| **rs7693777** | **4** | **40531393** | **[A/G]** | **1**  **0.9896** | **(60) 0.625**  **(58) 0.6105** | **(30) 0.3125**  **(32) 0.3368** | **(6) 0.0625**  **(5) 0.0526** | **0.3914**  **0.8268** | **0.2188**  **0.211** | **0** |
| **rs1017209** | **5** | **112872887** | **[T/C]** | **0.9896**  **1** | **(11) 0.1158**  **(19) 0.1979** | **(43) 0.4526**  **(42) 0.4375** | **(41) 0.4316**  **(35) 0.3648** | **0.9558**  **0.3173** | **0.3421**  **0.4167** | **2.25** |
| **rs244755** | **5** | **88131541** | **[A/G]** | **1**  **0.9896** | **(6) 0.0625**  **(5) 0.05226** | **(29) 0.3021**  **(29) 0.3053** | **(61) 0.6354**  **(61) 0.6421** | **0.3143**  **0.5199** | **0.2135**  **0.2053** | **0** |
| **rs2880075** | **6** | **149856351** | **[A/G]** | **1**  **1** | **(7) 0.0729**  **(6) 0.0625** | **(34) 0.3542**  **(39) 0.4063** | **(55) 0.5729**  **(51) 0.5313** | **0.5785**  **0.6796** | **0.25**  **0.2556** | **0.12** |
| **rs69521158** | **6** | **156169503** | **[T/C]** | **1**  **1** | **(4) 0.0417**  **(5) 0.0521** | **(35) 0.3646**  **(30) 0.3125** | **(57) 0.5938**  **(61) 0.6354** | **0.6252**  **0.5987** | **0.224**  **0.2083** | **0.14** |
| **rs10486722** | **7** | **41778433** | **[A/G]** | **0.9896**  **0.9896** | **(11) 0.1158**  **(6) 0.0632** | **(36) 0.3789**  **(32) 0.3368** | **(48) 0.5053**  **(57) 0.6** | **0.2865**  **0.5923** | **0.3053**  **0.2316** | **2.63** |
| **rs6952158** | **7** | **53238408** | **[A/G]** | **1**  **1** | **(24) 0.25**  **(23) 0.2396** | **(49) 0.5104**  **(50) 0.5208** | **(23) 0.2396**  **(23) 0.2396** | **0.8341**  **0.6769** | **0.4948**  **0.5** | **0** |
| **rs268599** | **8** | **71679955** | **[T/G]** | **0.9896**  **1** | **(10) 0.1053**  **(7) 0.0729** | **(32) 0.3368**  **(36) 0.375** | **(53) 0.5579**  **(52) 0.5521** | **0.1267**  **0.7912** | **0.2737**  **0.2604** | **0.05** |
| **rs6560224** | **9** | **73458543** | **[T/A]** | **1**  **1** | **(23) 0.2396**  **(28) 0.2917** | **(44) 0.4583**  **(40) 0.4167** | **(29) 0.3021**  **(28) 0.2917** | **0.4252**  **0.0956** | **0.4688**  **0.5** | **0.39** |
| **rs4877799** | **9** | **85511137** | **[A/G]** | **1**  **1** | **(15) 0.1563**  **(11) 0.1146** | **(47) 0.4896**  **(39) 0.4063** | **(34) 0.3542**  **(46) 0.4792** | **0.8486**  **0.529** | **0.401**  **0.318** | **3** |
| **rs6602883** | **10** | **47067933** | **[T/C]** | **1**  **0.9896** | **(5) 0.0521**  **(6) 0.0632** | **(39) 0.4063**  **(40) 0.4211** | **(52) 0.5417**  **(49) 0.5158** | **0.4924**  **0.5546** | **0.2552**  **0.2737** | **0.2** |
| **rs7903263** | **10** | **14738958** | **[A/C]** | **1**  **1** | **(4) 0.0417**  **(4) 0.0417** | **(40) 0.4167**  **(43) 0.4479** | **(52) 0.5417**  **(49) 0.5104** | **0.2665**  **0.1386** | **0.25**  **0.2656** | **0.12** |
| **rs11228758** | **11** | **56265052** | **[A/G]** | **1**  **1** | **(6) 0.0625**  **(10) 0.1042** | **(32) 0.3333**  **(41) 0.4271** | **(58) 0.6042**  **(45) 0.4688** | **0.572**  **0.8816** | **0.2292**  **0.3177** | **3.8** |
| **rs896618** | **11** | **40285758** | **[A/G]** | **1**  **1** | **(7) 0.0729**  **(4) 0.0417** | **(28) 0.2917**  **(28) 0.2917** | **(61) 0.6354**  **(64) 0.6667** | **0.1425**  **0.6691** | **0.2188**  **0.1875** | **0.58** |
| **rs2195238** | **12** | **99495702** | **[T/C]** | **1**  **1** | **(39) 0.4063**  **(46) 0.4792** | **(46) 0.4792**  **(44) 0.4583** | **(11) 0.1146**  **(6) 0.0625** | **0.6352**  **0.2746** | **0.3542**  **0.2917** | **1.72** |
| **rs2891405** | **12** | **111636480** | **[A/G]** | **1**  **1** | **(6) 0.0625**  **(7) 0.0729** | **(38) 0.3958**  **(30) 0.3125** | **(52) 0.5417**  **(59) 0.6146** | **0.7825**  **0.2482** | **0.2604**  **0.2292** | **0.51** |
| **rs292462** | **13** | **21912551** | **[G/C]** | **1**  **1** | **(17) 0.1771**  **(13) 0.1354** | **(40) 0.4167**  **(41) 0.4271** | **(39) 0.4063**  **(42) 0.4375** | **0.2283**  **0.5481** | **0.385**  **0.3494** | **0.55** |
| **rs951095** | **13** | **104276645** | **[T/C]** | **1**  **1** | **(57) 0.5938**  **(57) 0.5938** | **(31) 0.3229**  **(35) 0.3646** | **(8) 0.0833**  **(4) 0.0417** | **0.2054**  **0.6252** | **0.2448**  **0.224** | **0.23** |
| **rs214004** | **14** | **77875064** | **[T/A]** | **1**  **1** | **(50) 0.5208**  **(58) 0.6042** | **(39) 0.4063**  **(35) 0.3646** | **(7) 0.0729**  **(3) 0.0313** | **0.8695**  **0.3929** | **0.276**  **0.2135** | **2.03** |
| **rs1462266** | **14** | **97319155** | **[A/C]** | **1**  **1** | **(7) 0.0729**  **(4) 0.0417** | **(38) 0.3958**  **(31) 0.3229** | **(51) 0.5313**  **(61) 0.6354** | **0.9825**  **0.9799** | **0.2708**  **0.2031** | **2.43** |
| **rs2077596** | **15** | **25913330** | **[T/C]** | **1**  **0.9896** | **(30) 0.3125**  **(26) 0.2737** | **(49) 0.5104**  **(50) 0.5263** | **(17) 0.1771**  **(19) 0.2** | **0.6899**  **0.5594** | **0.4323**  **0.4632** | **0.37** |
| **rs2278295** | **15** | **50291434** | **[T/C]** | **1**  **1** | **(53) 0.5521**  **(57) 0.5938** | **(41) 0.4271**  **(29) 0.3021** | **(2) 0.0208**  **(10) 0.104** | **0.0574**  **0.04** | **0.2344**  **0.2552** | **0.23** |
| **rs1424144** | **16** | **69906999** | **[A/G]** | **1**  **1** | **(11) 0.1146**  **(10) 0.1042** | **(51) 0.5313**  **(33) 0.3438** | **(34) 0.3542**  **(53) 0.5521** | **0.2034**  **0.1617** | **0.3802**  **0.276** | **4.75** |
| **rs195867** | **16** | **26761626** | **[A/G]** | **0.9896**  **1** | **(4) 0.0421**  **(2) 0.0208** | **(35) 0.3684**  **(35) 0.3646** | **(56) 0.5895**  **(59) 0.6146** | **0.6027**  **0.207** | **0.2263**  **0.2031** | **0.3** |
| **rs8072552** | **17** | **36307042** | **[T/C]** | **1**  **1** | **(10) 0.1042**  **(12) 0.125** | **(40) 0.4167**  **(49) 0.5114** | **(46) 0.4792**  **(35) 0.3646** | **0.7619**  **0.4066** | **0.3125**  **0.3802** | **1.94** |
| **rs11655682** | **17** | **75778616** | **[T/G]** | **1**  **0.9896** | **(8) 0.0833**  **(8) 0.0842** | **(36) 0.375**  **(37) 0.3895** | **(52) 0.5417**  **(50) 0.5263** | **0.6132**  **0.7504** | **0.2708**  **0.2789** | **0.033** |
| **rs3893675** | **18** | **22339230** | **[T/C]** | **1**  **1** | **(11) 0.1146**  **(4) 0.0417** | **(34) 0.3542**  **(37) 0.3854** | **(51) 0.5313**  **(55) 0.5729** | **0.1531**  **0.4598** | **0.2917**  **0.2344** | **1.63** |
| **rs4131468** | **18** | **49727191** | **[T/A]** | **1**  **0.9792** | **(57) 0.5625**  **(57) 0.6064** | **(37) 0.3854**  **(36) 0.383** | **(5) 0.0521**  **(1) 0.0106** | **0.6715**  **0.0610** | **0.2448**  **0.2021** | **1** |
| **rs12459941** | **19** | **10527112** | **[T/C]** | **1**  **0.9896** | **(46) 0.4792**  **(59) 0.6211** | **(40) 0.4167**  **(29) 0.3053** | **(10) 0.1042**  **(7) 0.0737** | **0.7619**  **0.1995** | **0.3125**  **0.3363** | **3.6** |
| **rs29991** | **19** | **23332512** | **[T/C]** | **0.9896**  **1** | **(32) 0.3368**  **(19) 0.1979** | **(46) 0.4842**  **(52) 0.5417** | **(17) 0.1789**  **(25) 0.2604** | **0.9456**  **0.3811** | **0.4211**  **0.4688** | **4.65** |
| **rs1934915** | **20** | **36329274** | **[A/G]** | **1**  **1** | **(7) 0.0729**  **(11) 0.1146** | **(35) 0.3646**  **(29) 0.3021** | **(54) 0.5625**  **(56) 0.5833** | **0.6821**  **0.024** | **0.2552**  **0.2656** | **0.05** |
| **rs2183432** | **20** | **59728046** | **[A/G]** | **1**  **1** | **(53) 0.5833**  **(46) 0.4792** | **(33) 0.3438**  **(46) 0.4792** | **(7) 0.0729**  **(4) 0.0417** | **0.4822**  **0.0641** | **0.2448**  **0.2813** | **0.66** |
| **rs12482963** | **21** | **22894194** | **[A/G]** | **1**  **1** | **(34) 0.3542**  **(32) 0.3333** | **(46) 0.4792**  **(50) 0.5208** | **(16) 0.1667**  **(14) 0.1458** | **0.9462**  **0.4259** | **0.4063**  **0.4063** | **0** |
| **rs2833863** | **21** | **32808857** | **[A/G]** | **1**  **1** | **(7) 0.0729**  **(13) 0.1354** | **(41) 0.4271**  **(34) 0.3542** | **(48) 0.5**  **(49) 0.51** | **0.6547**  **0.0788** | **0.2865**  **0.3125** | **0.31** |
| **rs461915** | **22** | **21608658** | **[G/C]** | **1**  **1** | **(52) 0.5417**  **(53) 0.5521** | **(37) 0.3854**  **(37) 0.3854** | **(7) 0.0729**  **(6) 0.0625** | **0.9037**  **0.8899** | **0.2656**  **0.2552** | **0.05** |
