# Supplementary material for: Joint Effect of MCP-1 Genotype GG and MMP-1 Genotype 2G/2G Increases the Likelihood of Developing Pulmonary Tuberculosis in BCG-Vaccinated Individuals
Source: PLoS One. 2010 Jan 25;5(1):e8881. doi: 10.1371/journal.pone.0008881 (PMC2810343; doi:10.1371/journal.pone.0008881)
Supplement: Table S2 — Luciferase activity in response to PMA and M. tuberculosis-sonicate antigens stimulation of THP-1 cells transfected with vectors containing the -362 MCP-1 alleles G or C. A The promoter region spanning positions -537 to position -156 of MCP-1 gene containing the -362 MCP-1 alleles G and C were amplified from - 362 MCP-1 homozygous GG or CC and cloned into the Firely Luciferase vector pGL4.10[luc2] (Promega Corporation, Madison, WI), expanded in DH5α E. coli cells (Invitrogen, Carlsbad, CA) and purified using an endotoxin free plasmid purification system (Qiagen, Valencia, CA). B THP-1 cells were co-transfected with pGL4.10[luc2] vectors containing the -362 MCP-1 allele G or the allele C and control pGL4 Renilla Luciferase vector pGL4.73[hRluc/SV40] (Promega Corporation, Madison, WI) in a 50∶1 ratio using Effectene transfection reagents from Qiagen (Valencia, CA). Cells were cultured overnight in complete RPMI (10% FCS) and then washed three times in RPMI, re-suspended in complete RPMI, and stimulated overnight with or without PMA or M. tuberculosis-sonicate antigens. Protocols provided by the dual-luciferase assay system were followed for transfection of cells, preparation of cell lysates, and measurement of dual-luciferase signals. The data are presented as the ratio of Firefly Luciferase and Renilla Luciferase (control) signal. The standard deviation of means obtained from three independent experiments. C We run student t-tests using STATA10 to determine whether the alleles induce statistically different expression levels of luciferase in THP-1 cells. Vectors containing the -362 MCP-1 alleles are available upon request. (0.03 MB DOC) [file pone.0008881.s005.doc]

| ***MCP-1* -362 alleles**^A^ | **Luciferase activity + Standard deviation**^B^ | | |
| --- | --- | --- | --- |
|  | **No stimulation** | **PMA**  **(10 ng/ml)** | ***M. tuberculosis* sonicate**  **(10 ng/ml)** |
| *G* | 13.8 + 4 | 6.39 + 3 | 7.72 + 5 |
| *C* | 11.73 + 3 | 7.42 + 2 | 8.78 + 3 |
| Student t-test (allele *G* versus *C*)^C^ | Not significant | Not significant | Not significant |
